# Clinical Guidelines for Diagnosis and Treatment of Botulism, 2021

**DOI:** 10.15585/mmwr.rr7002a1

**Published:** 2021-05-07

**Authors:** Agam K. Rao, Jeremy Sobel, Kevin Chatham-Stephens, Carolina Luquez

**Affiliations:** 1 Division of Foodborne, Waterborne, and Environmental Diseases, National Center for Emerging and Zoonotic Infectious Diseases, CDC

## Abstract

Botulism is a rare, neurotoxin-mediated, life-threatening disease characterized by flaccid descending paralysis that begins with cranial nerve palsies and might progress to extremity weakness and respiratory failure. Botulinum neurotoxin, which inhibits acetylcholine release at the neuromuscular junction, is produced by the anaerobic, gram-positive bacterium *Clostridium botulinum* and, rarely, by related species (*C. baratii* and *C. butyricum)*. Exposure to the neurotoxin occurs through ingestion of toxin (foodborne botulism), bacterial colonization of a wound (wound botulism) or the intestines (infant botulism and adult intestinal colonization botulism), and high-concentration cosmetic or therapeutic injections of toxin (iatrogenic botulism). In addition, concerns have been raised about the possibility of a bioterrorism event involving toxin exposure through intentional contamination of food or drink or through aerosolization. Neurologic symptoms are similar regardless of exposure route. Treatment involves supportive care, intubation and mechanical ventilation when necessary, and administration of botulinum antitoxin. Certain neurological diseases (e.g., myasthenia gravis and Guillain-Barré syndrome) have signs and symptoms that overlap with botulism. Before the publication of these guidelines, no comprehensive clinical care guidelines existed for treating botulism. These evidence-based guidelines provide health care providers with recommended best practices for diagnosing, monitoring, and treating single cases or outbreaks of foodborne, wound, and inhalational botulism and were developed after a multiyear process involving several systematic reviews and expert input.

## Introduction

### Background

These evidence-based guidelines provide health care personnel with recommended best practices for diagnosing, monitoring, and treating botulism in the settings of conventional, contingency, and crisis standards of care. The following syndromes are described: foodborne botulism (exposure to botulinum neurotoxin in food), wound botulism (exposure to botulinum neurotoxin from a wound colonized with the bacteria), inhalational botulism (exposure to aerosolized botulinum neurotoxin, which could be caused intentionally), and iatrogenic botulism (exposure to botulinum neurotoxin by injection of high-concentration botulinum toxin for cosmetic or therapeutic purposes). These guidelines do not address syndromes of botulism caused by intestinal colonization by botulinum-toxin–producing Clostridia species (i.e., infant botulism and adult colonization botulism), which are inherently sporadic and have not occurred in outbreaks. Therefore, throughout the text, terms such as “botulism” and “patient with suspected botulism” refer to syndromes other than intestinal colonization.

Contamination of foods with botulinum neurotoxin can occur unintentionally when botulinum spores germinate under appropriate conditions and produce toxin, or intentionally, when toxin is added directly to foods. Foodborne botulism outbreaks usually affect few persons. However, because large outbreaks are possible (“epidemic potential”), foodborne botulism is a public health emergency. Contamination of wounds with *Clostridium botulinum* and subsequent in situ botulinum toxin production is typically (in the United States) caused by unsanitary injection of a particular type of heroin (black tar heroin) subcutaneously or subdermally; although common-source heroin containing clostridial spores might affect groups of injectors, wound botulism does not have the epidemic potential of foodborne botulism (*1*). Purified botulinum toxin, produced and weaponized by military biological warfare programs of various countries, could be dispersed as an aerosol and cause inhalational botulism. This form of botulism, which does not occur naturally and has been reported once in a laboratory worker, could affect many persons (*2*).

Diagnosis of botulism depends on high clinical suspicion and a thorough neurologic examination. The timeliness of diagnosis is crucial to successful treatment because botulinum antitoxin, the only specific therapy for botulism, must be administered to patients as quickly as possible. In the United States, botulinum antitoxin (to treat suspected botulism, other than infant botulism) is available emergently and free of charge from the federal government. Health departments and CDC provide emergency clinical consultations 24 hours per day and facilitate rapid antitoxin delivery for treatment of suspected botulism, other than infant botulism. For suspected cases of infant botulism, the California Department of Public Health Infant Botulism Treatment and Prevention Program provides clinical consultation and access to the specific antitoxin licensed for treatment of infant botulism.

The recommendations in these guidelines address the conventional standard of care, in which medical resources are not limited, as well as settings of contingency and crisis standards of care, with limited medical resources. These guidelines focus on clinical management in the acute phase of illness and do not address long-term care, epidemiologic investigations, antitoxin for postexposure prophylaxis, and management of routine medical issues that are not specific to botulism. Clinicians, hospital administrators, state and local health officials, and planners can use the recommendations in these guidelines to assist in developing crisis protocols for national preparedness for botulism events ranging from sporadic (single) cases to large outbreaks.

### Pathophysiology of Botulism

Botulism is caused by toxins formed by the anaerobic, gram-positive bacterium *C. botulinum* and, rarely, by strains of closely related species (*C. baratii* and *C. butyricum)* (*3*). These organisms form spores that are ubiquitous in the environment and capable of indefinitely surviving most naturally occurring conditions as well as boiling and other routine cooking practices. Spores are routinely ingested by humans but do not normally germinate in the intestine (*4*). Toxin is produced only when the spores germinate; this occurs under a rare confluence of circumstances that include anaerobic conditions, low acidity (pH >4.5), low salt and sugar content, and temperatures of 37°F–99°F (3°C–37°C), depending on the serotype. Botulinum toxins are the most potent biologic toxins known. Although the precise lethal dose for humans is unknown, extrapolations have been made from primate studies. The lethal doses for purified crystalline botulinum toxin type A for a 154-lb (70-kg) man are estimated to be 70 *μ*g when introduced orally and 0.80–0.90 *μ*g when inhaled (*2*). Lower doses were proposed in older studies (*5*–*7*).

Seven antigenically distinct botulinum toxins have been identified (A, B, C, D, E, F, and G), all during 1919–1970 (*3*); most strains of *C. botulinum* produce only a single toxin, although strains producing two toxin types have been identified (*8*). In addition, two novel botulinum-toxin–like proteins have been identified from gene sequences and assembled: one from a *C. botulinum* isolate and one from an *Enterococcus faecium* isolate (*9*–*11*). All botulinum toxin types share a similar structure, consisting of a zinc-endopeptidase protein formed by a heavy chain of approximately 100,000 daltons and a light chain of approximately 50,000 daltons. Botulinum neurotoxin enters the vascular circulation (through ingestion, absorption from colonized wound or intestine, inhalation, or injection) and is transported to peripheral cholinergic nerve terminals, including neuromuscular junctions, postganglionic parasympathetic nerve endings, and peripheral ganglia (*12*). All toxin types produce a similar clinical syndrome of cranial nerve palsies followed by descending symmetric flaccid paralysis of variable severity and extent through similar pharmacological mechanisms at the neuromuscular junction (*12*–*14*).

The sequence of botulinum neurotoxin activity at the neuromuscular junction includes heavy-chain binding to a neuronal cell followed by internalization by means of receptor-mediated endocytosis, translocation to the cytosol, and cleavage of the proteins (specific for each serotype) involved in the release of the neurotransmitter acetylcholine (*4*). The characteristic flaccid paralysis results from blocking acetylcholine transmission across the neuromuscular junction by inhibition of acetylcholine release from the presynaptic motor neuron terminal (*12*). The large molecular size of the botulinum toxin likely precludes its crossing the blood-brain barrier to the central nervous system (*4*). Botulinum toxin might be transported to the central nervous system axonally, similar to tetanus toxin, which it resembles, although direct effects on the central nervous system have not been documented in humans (*15*). Recovery, which takes weeks to months, occurs after sprouting of new nerve terminals.

Toxin serotypes A, B, E, and (more rarely) F cause human disease. Toxin type A produces the most severe syndrome, with the highest proportion of patients requiring mechanical ventilation (*14*,*16*,*17*). Toxin type B usually causes milder disease than type A (*14*,*16*,*17*). Only two cases of illness in humans from toxin type C and one outbreak caused by toxin type D have been reported, all in the 1950s (*18*,*19*). Although toxin type C blocks neuromuscular transmission in human tissue in laboratory experiments, this toxin type might not be absorbed in the human gastrointestinal tract (*20*,*21*). In studies of human tissue, toxin type D has been reported not to block neuromuscular transmission (*20*). No cases in humans have been reported from toxin type G (*3*). Toxin type E, usually associated with consumption of foods of aquatic origin, produces a syndrome of variable severity, which frequently includes gastrointestinal symptoms (*17*,*22*,*23*). Type F cases are rare and characterized by rapid progression, extensive paralysis, and respiratory failure but with earlier recovery (*24*,*25*). All toxin types readily produce botulism in experimental animal models.

## Methods

In 2015, CDC established a technical development group with experts in the clinical, epidemiologic, and laboratory aspects of botulism and related fields. To oversee and guide development of the guidelines, CDC also established an internal agency guideline steering committee comprising clinical, preparedness, and response experts. Together, the technical development group and the guidelines steering committee prioritized topic areas and determined the scope of the guidelines. Priorities included characterizing the clinical features of botulism, determining optimal monitoring of patients with suspected botulism, and establishing the efficacy and safety of botulinum antitoxin (*26*). Children and pregnant women were considered specifically. How botulism might uniquely affect these populations was considered throughout the process, including through systematic reviews on pediatric botulism and botulism in pregnant women, as well as presentations during two forums and a workshop. A committee comprising representatives from federal agencies, including the Assistant Secretary for Preparedness and Response, the Biomedical Advanced Research and Development Agency, the U.S. Department of Homeland Security, the U.S. Department of Defense, the Food and Drug Administration (FDA), and the National Institutes of Health, was briefed throughout the process of developing the guidelines. 

Physicians from CDC and academia conducted six systematic reviews on the clinical features of botulism, pediatric botulism, botulism in pregnancy, epidemiology of botulism outbreaks, botulinum antitoxin efficacy, and allergic reactions to botulinum antitoxin (*14*,*27*–*31*). The systematic reviews were conducted in accordance with the Preferred Reporting Items for Systematic Reviews and Meta-Analyses (PRISMA) statement (http://www.prisma-statement.org) and registered with the International Prospective Register of Systematic Reviews (Table 1). CDC librarians developed the search strategy for each review, using search terms selected in consultation with the review authors. The librarians searched the following databases from inception through May or November 2015: ClinicalTrials.gov (all trials indexed through May or November 2015), Cochrane Library (1800–present), Cumulative Index to Nursing and Allied Health Literature EBSCO (1981–present), Embase Dialog (1947–1988), Embase Ovid (1988–present), Global Health Ovid (1910–present), Medline Ovid (1946–present), and Scopus (1960–present). For the clinical features, pediatric, and antitoxin efficacy systematic reviews, librarians also searched the National Technical Information Service, Defense Technical Information Center (DTIC), and government documents via WorldCat and DTIC documents via Google Scholar. Two authors of the clinical features systematic review also manually searched *Morbidity and Mortality Weekly Reports (MMWRs)* published before 1980. The allergy systematic review included unpublished data from Cangene Corporation from two clinical trials involving 56 healthy adults and from CDC’s botulism consultation service for 249 patients with botulism treated with botulinum antitoxin heptavalent (BAT). The antitoxin efficacy systematic review included animal studies that reported controlled toxin exposure and antitoxin treatment. The pediatric and pregnancy systematic reviews included non–English-language articles that were professionally translated; the other systematic reviews included only English-language articles. The six systematic reviews were published, along with nine other manuscripts on various botulism topics, including clinical characteristics and ancillary test results among patients with botulism and clinical criteria to trigger suspicion for botulism, that also informed these guidelines (*13*,*26*,*32*–*38*). In addition, an analysis of detailed abstractions of medical records from 99 hospitalized patients with laboratory-confirmed botulism provided data on presence and progression of signs and symptoms (CDC, unpublished data, 2016).

**TABLE 1:** Botulism clinical guidelines topic areas and questions for systematic reviews

Topic area	Questions
Maternal and fetal outcomes associated with botulism	Are pregnant and postpartum women more susceptible than nonpregnant women to botulism? Do pregnant women have different signs and symptoms or more severe disease than nonpregnant patients? Is there an increased risk for adverse maternal, fetal, or neonatal outcomes associated with botulism? What are the effects of antitoxin on pregnant women?
Allergic reactions to botulinum antitoxin	What is the risk for anaphylaxis from botulinum antitoxin? What is the usefulness of skin testing in determining the risk for allergic reactions to botulinum antitoxin?
Efficacy of antitoxin in foodborne botulism	What are the benefits of botulinum antitoxin? Is there a time beyond which antitoxin is no longer beneficial? Do any patient demographic or clinical characteristics predict greater benefit from antitoxin?
Pediatric botulism and use of botulinum antitoxin in children	What are the signs and symptoms of diagnostic value in children with botulism? What is the effect of botulinum antitoxin in children?
Epidemiology of foodborne botulism outbreaks	What are the demographic characteristics? What are the types of food sources and toxin types? What are the clinical characteristics, including adverse outcomes? What are the times between exposure, symptom onset, and adverse outcomes? What are the outbreak durations?
Clinical features of foodborne and wound botulism	What are the signs and symptoms reported at hospital admission? What are the incubation periods and duration of illness before hospital admission? What are the patient factors associated with respiratory failure and death?

In partnership with the National Association of County and City Health Officials, CDC organized two forums conducted during January–May 2016 to elicit the individual input of experts in botulism, crisis standards of care, and clinical medicine. One forum focused on the diagnosis and management of botulism (10 teleconferences with 16 experts) and the other on the treatment of botulism (11 teleconferences with 21 experts). Data from the systematic and other reviews, individual published and unpublished studies, CDC investigations, and the relevant botulism, pharmacologic, respiratory, neurologic, and critical care literature were presented to the experts in each of the forums. Experts represented themselves and provided their own input on various topics, including botulism pathophysiology, infectious diseases, antitoxin use, emergency and critical care medicine, crisis standards of care, electrodiagnostic testing, public health, obstetrics and gynecology, and pediatrics.

CDC used input from the two forums to generate preliminary recommendations for a 2-day botulism workshop in June 2016. A total of 72 experts, including the technical development group, steering committee, most of the expert forum participants, and other invited participants, provided their own input on targeted questions posed by CDC about the diagnosis and management of botulism. Participants included physicians who have treated patients with botulism, representatives from professional societies (e.g., the American College of Emergency Physicians), state and federal public health officials, and botulism subject matter experts. Participants provided their own input in response to CDC solicitation throughout the process. CDC staff and other participants presented the evidence compiled from the systematic reviews and forums and gave presentations on various topics, including crisis standards of care, pathophysiology of botulism, caring for patients with botulism during an outbreak, ethical considerations in the management of botulism, antitoxin safety and effectiveness, and considerations for vulnerable populations. Of note, the peer-reviewed scientific literature on clinical aspects of botulism consists largely of case series and case reports.

CDC asked participants to declare any potential conflict of interest. Two experts reported such potential conflicts. CDC reviewed these potential conflicts of interest and determined that they did not preclude these persons from participating. The authors of the guidelines have no financial interests in or other competing interests with the manufacturers of commercial products or suppliers of commercial services related to botulism. After the workshop, the authors wrote a draft of the guidelines based on the systematic reviews and forum and workshop discussions. The draft was reviewed by selected members of the technical development group and steering committee and then sent to workshop participants for their individual review. The recommendations presented in this report reflect the synthesis and analysis of evidence obtained from systematic reviews conducted by CDC scientists and input from subject matter experts. These guidelines will be updated when substantive new evidence is available regarding the diagnosis, monitoring, and treatment of foodborne, wound, inhalational, or iatrogenic botulism.

## Standards of Care: Conventional, Contingency, and Crisis

The acute onset of neuromuscular weakness in patients with botulism, frequently progressing to respiratory failure, typically requires high acuity emergency and inpatient care. Therefore, a sudden influx of severely ill patients with botulism might stress the ability of a single hospital or a hospital network to provide appropriate care. To prepare for potentially catastrophic events such as botulism outbreaks, the Institute of Medicine (National Academies of Medicine) recommends that officials from state and local governments (e.g., public health and emergency management), emergency medical services, and health care organizations establish disaster response plans (*39*). These plans should incorporate crisis standards of care to optimize medical surge capacity and guide the process of medical care during a catastrophic event.

The medical surge capacity continuum is determined by the balance between health care supply and demand. The standards of care within this continuum are categorized as conventional, contingency, or crisis (*39*).

Components of the health care supply are space (e.g., rooms or areas in which to care for patients), staff (e.g., health care providers), supplies (e.g., medications and medical supplies such as tongue blades), and equipment (e.g., ventilators and monitors). In the conventional (i.e., standard) care setting, standard clinical care spaces, staff, and supplies are used, resulting in a usual level of patient care. In the contingency care setting, although substitutions of space, staff, or supplies are required as demand increases, the level of patient care is not affected. In the crisis care setting, the demand for space, staff, or supplies exceeds that which is available, affecting the level of patient care that can be provided. For example, in a hospital that is providing a conventional standard of care, managing an increasing number of patients might include adding beds to patient rooms to treat more patients. In contingency standard of care settings, measures might include caring for patients in areas not typically used for inpatient care (e.g., postanesthesia care unit and endoscopy suite), and measures in crisis standard of care settings might include caring for patients in areas that are never used for patient care (e.g., classrooms) (*40*). In a crisis standard of care setting, the focus transitions from actions that prioritize individual patient outcomes to actions that prioritize population-based outcomes. Implementation of crisis standards of care in a given facility should be as brief as possible, and every effort should be made to either obtain appropriate resources or transfer patients to appropriately resourced facilities so conventional standards of care can be resumed.

Disaster response plans should incorporate indicators to measure or predict demand for health care services or resources (e.g., emergency department wait time) and triggers that guide decisions about delivering those health care services or resources. For example, an emergency department wait time of greater than a specified period might result in increased staffing (*41*). These indicators can mark the transition from conventional to contingency and from contingency to crisis, and the triggers describe which actions should be taken in response to the indicators. Indicators and triggers during a botulism outbreak likely will vary across hospitals (*40*). For example, in some hospitals, a conventional standard of care might be appropriate in the emergency department that has five patients in respiratory distress with possible botulism; however, a contingency or crisis standard of care might be needed for other hospitals. Event-specific criteria that might need to be considered in a botulism outbreak include the number of patients affected, the severity of their illness (e.g., patients requiring intubation and mechanical ventilation), and availability of botulinum antitoxin and mechanical ventilators. Certain aspects of a botulism outbreak response, including diagnosis, monitoring, and treatment, might vary across the medical surge capacity continuum (conventional, contingency, and crisis standards of care) (Table 2).

**TABLE 2:** Conventional, contingency, and crisis standards of care for diagnosing, monitoring, and treating botulism*

Definition, setting, and treatment	Standard of care		
	Conventional	Contingency	Crisis
Definition	Usual standard of care, and space, staff, supplies, and equipment are available.	Care is the same as in conventional settings but might involve different methods, medications, or locations; the impact on usual standard of care is minimal.	Critical space, staff, supplies, or equipment are limited, affecting usual standard of care and requiring medical care prioritization. Care might not be initiated and might be withdrawn from persons to allow resources to be allocated to persons with the highest likelihood of survival or benefit. Because prognosis in botulism is excellent with appropriate respiratory support, airway control, and ventilation, transfer to an adequately resourced facility should be attempted when at all possible.
Hospitals	Usual patient care areas are used. Physicians and advanced practice providers diagnose botulism based on history, examination, and laboratory tests. Consult public health officials immediately when botulism is suspected, and request antitoxin. Triage based on severity of illness and respiratory status. Admit all patients with suspected botulism to the appropriate unit in which close neurologic and respiratory monitoring is available. If antitoxin is available and a patient needs to be transferred to a higher acuity hospital, consider administering antitoxin before transfer and ensure serial monitoring can be performed while in transit. Conduct full diagnostic testing, including neurologic examination, brain imaging, lumbar puncture, electromyography, and nerve conduction study as applicable. Perform serial monitoring with a complete neurologic examination, including cranial nerves, extremity strength, and respiratory status, before and after antitoxin administration. Monitor for adverse events (e.g., anaphylaxis) during and after antitoxin administration.	Critical care surge plans are implemented; use adjunct areas (e.g., procedure rooms). Physicians and advanced practice providers diagnose botulism based on history, examination, and laboratory tests. Consult public health officials immediately when botulism is suspected, and request antitoxin. Triage based on severity of illness and respiratory status. Admit patients with suspected botulism requiring hospitalization (e.g., patients with respiratory symptoms or difficulty swallowing). If antitoxin is available and a patient needs to be transferred to a higher acuity hospital, consider administering antitoxin before transfer, and ensure serial monitoring can be performed while in transit. Conduct more limited testing and evaluation using the clinical criteria tool for early diagnosis of botulism. Perform serial monitoring using the clinical criteria tool for early diagnosis of botulism to identify illness progression Monitor for adverse events (e.g., anaphylaxis) during and after antitoxin administration.	The maximal critical care surge plan is implemented; use all available areas (e.g., procedure rooms). All medical staff (e.g., physicians and nurses) diagnose botulism using the clinical criteria tool for early diagnosis of botulism. Consult public health officials immediately when botulism is suspected, and request antitoxin. Triage based on severity of illness and respiratory status. Admit patients with suspected botulism requiring hospitalization based on current capacity (e.g., patients with respiratory symptoms or difficulty swallowing). If antitoxin is available and a patient needs to be transferred to a higher acuity hospital, consider administering antitoxin before transfer and ensure serial monitoring can be performed while in transit. For patients not requiring hospitalization, refer stable, moderately ill patients to alternate care sites and send stable, mildly ill patients home (ensure connection with public health resources for telephone check-ins, and provide list of symptoms to self-monitor). Further limit testing and evaluation subject to resource availability (e.g., limit lumbar punctures, electrodiagnostic testing, and neuroimaging). Perform serial monitoring focused on illness progression, ability to swallow, and respiratory status for patients who do not require intubation. Monitor for adverse events (e.g., anaphylaxis) during and after antitoxin administration.
Medical facilities that are not hospitals	Refer patients with suspected botulism to the hospital.	Refer patients with suspected botulism to the hospital. For exposed persons without signs or symptoms of botulism, consider observing on site or asking them to self-monitor at home for signs or symptoms consistent with botulism and go to the hospital if symptomatic. Discharge asymptomatic persons with unknown exposure home to self-monitor.	Refer severely ill patients with suspected botulism to the hospital. Send mildly ill patients who do not require hospitalization home to self-monitor for signs and symptoms with telephone follow-up. Consider locally established alternate care sites (e.g., federal medical stations) to provide for overflow and convalescent care to augment hospitals. Discharge concerned, asymptomatic persons with unknown exposure home to self-monitor.
Treatment with antitoxin	Consider treatment with antitoxin of any patient with suspected botulism. Patients with mild symptoms, reliably observed to have no progression of paralysis over time, might not require treatment.	Prioritize treatment of patients with features most suggestive of botulism. Use antitoxin with the goal of preventing respiratory collapse requiring mechanical ventilation; prioritize patients with progressing paralysis who are not likely to require intubation before antitoxin can be administered.	Prioritize treatment of patients with features most suggestive of botulism. Use antitoxin with the goal of preventing respiratory collapse requiring mechanical ventilation; prioritize patients with progressing paralysis who are not likely to require intubation before antitoxin can be administered.

* Sources: Institute of Medicine. Crisis standards of care: a systems framework for catastrophic disaster response. Washington, DC: The National Academies Press; 2012; and Hick JL, Barbera JA, Kelen GD. Refining surge capacity: conventional, contingency, and crisis capacity. Disaster Med Public Health Prep 2009;3(Suppl 2):S59–67.

## Diagnosis of Botulism

Each of the following sections begins with a summary of evidence. Following the evidence are the CDC recommendations for diagnosing, monitoring, and treating suspected botulism as well as (in certain sections) key points for clinicians. In addition, a summary of the recommendations and key points is provided (Supplementary Box; https://stacks.cdc.gov/view/cdc/105129).

Botulism typically produces a distinctive syndrome of cranial nerve palsies that can be followed by bilateral, symmetric, descending flaccid paralysis, affecting proximal before distal limb musculature, that might progress to respiratory failure and death. The extent and severity of paralysis is proportional to the dose of toxin. Patients with botulism are described as alert and oriented, although ptosis, ocular muscle paralysis, voice changes from vocal cord paralysis, and gait disturbance from skeletal muscle paralysis can be mistaken as manifestations of drug or alcohol intoxication or mental status changes of other origin; patients rarely have sensory deficits and rarely report pain (*3*,*42*,*43*). However, the diagnosis of botulism is frequently delayed or even missed.

### Challenges in the Diagnosis of Botulism

Although the progression of paralysis in patients with botulism is described as unique and recognizable, in practice, when a patient is first seen by the health care provider, the neurologic symptoms and the sequence of progression both are sometimes misdiagnosed (*3*,*14*). The reasons for initial failure to diagnose botulism in subsequently confirmed cases has been investigated most productively in outbreaks, in which cases initially misdiagnosed were eventually identified by outbreak investigators. Outbreak investigations in which some botulism cases were only identified after the patients had been discharged with alternative diagnoses highlight the potential for delayed or missed diagnoses (*35*,*38*) (CDC, unpublished data, 2016). The critical initial treatment and management decisions for patients with suspected botulism must be made based on clinical findings. Botulinum antitoxin, the only specific therapy for botulism, should be administered as quickly as possible. Laboratory confirmation can take several days, and delaying administration of antitoxin to a patient with a high or medium likelihood of botulism while awaiting laboratory results can worsen the patient’s outcome (*3*,*35*,*44*,*45*). The diagnostic challenges resulting from the variations in the spectrum of signs and symptoms of botulism were highlighted in the delayed recognition of a large foodborne botulism outbreak, in which some patients initially received diagnoses of myasthenia gravis, stroke, or psychiatric disorders (*46*). Most of the affected patients were reported to have had the classic signs and symptoms of botulism (*46*). In recent literature reviews and a classical case series, botulism was most commonly misdiagnosed as myasthenia gravis and Guillain-Barré syndrome (*13*,*14*,*16*,*30*,*31*). A wide variety of common and unusual etiologies have been included in differential diagnoses of individual cases (e.g., cerebrovascular accident, Lambert-Eaton syndrome, meningitis, encephalitis, and tick paralysis) (*13*). In a review of 332 patients with possible botulism for which CDC consulted during 1980–2016, the treating physician provided alternate diagnostic considerations for 274 cases (83%); for these, treating physicians reported a range of zero to six illnesses other than botulism as possible diagnoses at the time of emergency public health consultation. The most common differential diagnoses were Guillain-Barré syndrome (99 cases) and myasthenia gravis (76 cases). For 160 botulism cases that occurred during 2009–2015, treating physicians who consulted with the CDC botulism clinical consultation service listed botulism first for 90% (144) of cases, second for 6% (10), third for 3% (five), and fourth for <1% (one).

In children and adolescents, a differential diagnosis was reported in 79 (22%) cases; the most commonly listed alternate diagnoses were myasthenia gravis (22 cases; 28%), poisonings and intoxications (20 cases; 25%), Guillain-Barré syndrome (11 cases; 14%), and poliomyelitis (nine cases; 11%) (*31*). Misdiagnosis of botulism, including in outbreak settings, might occur because botulism is much less common than other diseases with similar signs and symptoms, such as myasthenia gravis and Guillain-Barré syndrome. In addition, failure to perform a thorough neurologic examination and identify the typical neurologic findings might decrease the likelihood of considering botulism (*26*). Although rarely reported, atypical findings or progression, such as reported asymmetry of deficits, might explain diagnostic difficulties (*33*,*34*,*38*,*47*).

### Signs and Symptoms

In reviews and analyses conducted while gathering evidence for these guidelines (*13*,*14*,*16*,*30*,*31*,*36*), the most commonly reported symptoms among patients with botulism were dysphagia; blurred vision; slurred speech, difficulty speaking, and hoarse voice; gastrointestinal symptoms; dry mouth; shortness of breath; and diplopia. The most common signs were descending paralysis, ptosis, and ophthalmoplegia.

Signs and symptoms of botulism evolve over a period of hours to a few days. Initially, subjective symptoms of minor visual changes or (in patients with foodborne botulism) abdominal discomfort might occur, followed by progressive cranial palsies, which might then be followed by descending flaccid bilateral paralysis. In different patients, the maximum extent of neurologic signs might range from only ptosis or mild cranial nerve findings to descending bilateral flaccid paralysis, encompassing cranial nerve–innervated respiratory, extremity, and axial muscles. Early gastrointestinal symptoms (e.g., nausea and vomiting) are more common among persons with foodborne botulism than with other types of botulism (*13*,*14*,*16*). For example, vomiting was reported by 172 (50%) patients with foodborne botulism compared with three (5%) with wound botulism (*14*). Whether gastrointestinal signs and symptoms are caused by botulinum neurotoxin, other clostridial products, or nonclostridial substances related to food spoilage is unknown. Whether foodborne botulism from intentional contamination of food with purified botulinum toxin would cause gastrointestinal signs and symptoms is unknown (*14*). Constipation is often reported as an early symptom among children (*31*). Infants and young children might not be able to describe symptoms such as double vision; signs were more commonly reported than symptoms among children (*31*). The terminology used to describe the neurologic manifestations of botulism in infants differs from that used for children and adolescents; for example, in one analysis, hypotonia, weak cry, and poor feeding were reported among the three infants with foodborne botulism but not in other children (*36*).

Botulism is typically described as producing symmetric neurologic deficits, and the pathophysiological mechanism of the disease (i.e., circulatory distribution of the toxin to neuromuscular junctions) (*12*) is consistent with this description. Certain detailed case studies describe individual patients with asymmetric neurologic deficits (*47*). Larger case series have reported asymmetry or unilateral neurologic deficits in the range of 6%–15% of patients (*13*,*16*). These data are difficult to interpret because many case series present the findings from clinical chart abstractions; data in charts might be missing or incomplete and are typically reported by multiple providers with varying levels of expertise and charting habits. Unreactive pupils are expected in patients with botulism but were reported in only 25% in a large series of confirmed cases (i.e., cases with a positive specimen or epidemiologic association of a clinically compatible case with a case with a positive specimen) (*13*). Although rare, symptoms such as fever, nondescending paralysis, and altered mental status have been reported (*13*,*31*). Botulism typically affects proximal before distal muscles; however, equal muscle strengths or even distal muscles weaker than proximal have been reported (*13*). The reasons for these rarely reported findings might include an inadequately performed or recorded neurologic examination, a preexisting focal deficit, coincident processes such as infection, or a rare variance from the classical syndrome.

Respiratory failure without preceding neurologic deficits has rarely been reported as the presenting symptom. Such a presentation is highly improbable and likely represents an inability to perform a timely, thorough neurologic examination, which would have revealed the cranial nerve palsies that precede pharyngeal compromise and respiratory muscle paralysis.

In a series of 72 patients with sporadic (i.e., single) cases of botulism, most had a chief complaint that included symptoms reflecting the classical neurologic deficits of botulism (i.e., slurred speech, weakness, and difficulty swallowing). However, some of the primary signs and symptoms were less indicative of botulism (e.g., gastrointestinal symptoms only, back pain and difficulty using a walker, altered consciousness, and lip and tongue numbness) (CDC, unpublished data, 2016). Patients whose primary signs and symptoms did not reflect classical neurologic deficits of botulism were more likely to have a delayed diagnosis of botulism (CDC, unpublished data, 2016).

### Key Points for Clinicians

- Be aware of the spectrum of signs and symptoms of botulism, ranging from limited cranial nerve palsies (e.g., ptosis) to respiratory failure and complete extremity paralysis.
- Be aware that the respiratory system might be compromised early in the illness, when respiratory muscles (e.g., diaphragm) are unaffected but the upper airway is compromised from paresis of cranial nerve muscles, resulting in pharyngeal collapse or pooling of secretions.

### Recommendations

- Consider botulism when myasthenia gravis or Guillain-Barré syndrome are suspected and in a patient with unexplained symmetric cranial nerve palsies, with or without paresis of other muscles.
- Conduct thorough, serial neurologic examinations to detect the neurologic deficits of botulism and their progression.
- If botulism is suspected, immediately contact the local or state health department’s emergency on-call staff to arrange an emergency expert clinical consultation and, when indicated, request botulinum antitoxin from CDC.

### Ancillary Testing

#### Background

Results from routine laboratory tests, including complete blood counts, examination of cerebrospinal fluid (CSF), and radiologic studies, are typically normal in patients with botulism. In Guillain-Barré syndrome, CSF protein concentrations are often elevated, especially by the second week of illness (*48*). In patients with botulism, mild increases in CSF protein concentrations are not reported frequently (*13*). Brain imaging might help exclude brainstem strokes that can produce nonlateralizing symptoms. The Tensilon (edrophonium) test, historically used to help diagnose myasthenia gravis, is usually negative in patients with botulism, although minimal responses have been reported (*36*).

Electrodiagnostic studies such as repetitive nerve stimulation (RNS), electromyography (EMG), and nerve conduction studies (NCSs) can help elucidate the etiology of muscle weakness. RNS involves electrically stimulating a motor nerve at either low (2–3 Hz or possibly 5 Hz) or high frequency (30–50 Hz) and recording the response in the distal muscle. EMG involves inserting a needle electrode into a muscle and recording the electrical activity at rest and with effort, showing motor unit potentials or motor unit action potentials. An NCS involves providing an electrical stimulus to a nerve and recording the electrical response from a sensory nerve (sensory nerve conduction study) or muscle (motor nerve conduction study) (*49*). Distinctive classical findings of botulism are an increment in the compound motor nerve action potential amplitude, with RNS rates of 30–50 Hz (*50*); fibrillation; decreased recruitment of muscle units; decreased duration of muscle unit potentials with EMG; and decreased motor-evoked amplitude on an NCS with otherwise normal findings (*49*). However, early in the disease course, electrodiagnostic studies might be normal or almost normal and therefore not helpful.

EMG, RNS, and NCSs have several limitations. They are operator-dependent and technically challenging, require specialized training and equipment, are not available at all hospitals, and can take 2 hours to complete; in addition, the results require expert interpretation. Early in the course of botulism, electrodiagnostic testing results are likely to be normal (except for testing by single-fiber EMG) (*51*,*52*); late in the course, abnormalities are detected by these tests. EMG requires cooperation from the patient. The entire examination can be painful, especially RNS and particularly at 30–50 Hz (*49*). Clinicians must remember that patients with botulism who are paralyzed and intubated are still conscious (unless they are sedated); therefore, they should explain to patients who are not sedated why electrodiagnostic testing is being conducted and what they should expect. The sensitivity and specificity of EMG, RNS, and NCSs for diagnosing botulism are unknown. Electrodiagnostic findings in patients with other neuromuscular diseases (e.g., the Miller Fisher variant of Guillain-Barré syndrome) can be similar to those of botulism (*50*,*51*). More focused EMG studies, such as single-fiber EMG with measurement jitter, might be more sensitive (although it is nonspecific) than the general EMG but require even more specialized training, more expensive equipment, and more cooperation from the patient (*52*). The findings of electrodiagnostic studies should always be considered in the context of clinical, epidemiologic, and laboratory data.

Results from electrodiagnostic studies might help with the diagnosis of suspected botulism in settings of conventional, contingency, and crisis standards of care, depending on the situation. During an outbreak, electrodiagnostic studies are rarely needed for a cluster of patients with a clear history of bilateral, symmetric cranial nerve palsies followed by descending paralysis. However, for patients for whom the diagnosis is not clear, electrodiagnostic studies might help distinguish botulism from other neuromuscular diseases (e.g., myasthenia gravis or Guillain*-*Barré syndrome). This is especially true for sporadic (single) cases, in which increasing the diagnostic certainty of botulism as early as possible in the course of illness helps guide clinicians in making the critical decision to treat with antitoxin for suspected botulism rather than plasmapheresis or immunoglobulin therapy for suspected Guillain*-*Barré syndrome. Because findings from electrodiagnostic studies might remain abnormal for weeks after illness onset, these studies might be useful in the later stages of illness, when botulinum toxin is unlikely to be detectable in the serum. Whereas the identification of multiple patients with cranial nerve palsies and descending flaccid paralysis is highly suggestive of an outbreak of botulism, the additional evidence from electrodiagnostic studies can provide support for clinical and public health management decisions. Electrodiagnostic studies were helpful in establishing the diagnosis of botulism in an outbreak with patients who demonstrated atypical features (CDC, unpublished data, 2015). For public health events that require contingency or crisis standards of care, the likelihood of being able to conduct electrodiagnostic studies decreases.

#### Recommendation

- When feasible, consider using electrodiagnostic testing to assist in diagnosis of a suspected botulism case. When conducted and interpreted by experts, EMG, RNS, and NCSs can provide useful diagnostic data.

### Exposure Risk Factors and Botulism Diagnosis

#### Background

A known risk factor for botulism in a patient’s clinical history can help focus the clinician’s attention on the diagnosis. Risk factors for wound botulism include injection drug use (especially of black tar heroin) and for foodborne botulism include consumption of home-canned food (*3*). However, because atypical and novel exposures also result in botulism, the absence of typical exposure risk factors does not rule out the disease. The occurrence of more than one case of illness that is suspected to be botulism, especially among persons with some connection to one another, suggests a common-source outbreak and substantially increases the likelihood of the diagnosis (*3*). However, the occurrence of geographically dispersed cases among persons with no obvious connection does not rule out the possibility of a botulism outbreak that could be caused by a widely distributed, seemingly innocuous product. Public health authorities should immediately investigate all cases of suspected botulism and, when exposures are suspected, inform clinicians about them promptly so that other patients with compatible signs and symptoms can be interviewed and possibly linked to the outbreak.

#### Recommendation

- Clinicians should ask patients about possible exposures to well-described sources of botulinum toxin, while keeping in mind that absence of such exposures does not exclude the possibility of botulism.

### Clinical Criteria Tool for Early Diagnosis of Botulism in Crisis and Contingency Settings

**FIGURE 1:**
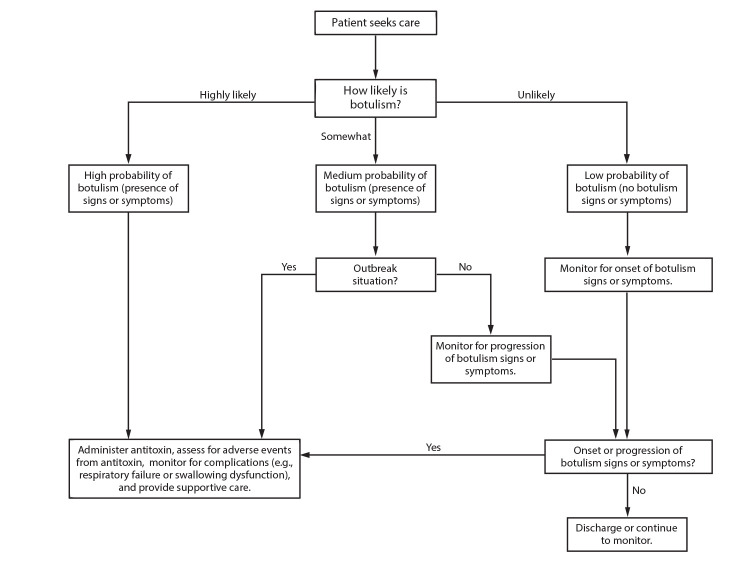
Assessing patients with known or possible exposure to botulinum toxin in conventional and contingency settings* * When assessing the likelihood of botulism, consider clinical criteria and available epidemiologic data. Classify patients into a botulism likelihood category per the clinician’s judgement. Additional information on clinical criteria is available (Rao AK, Lin NH, Griese SE, Chatham-Stephens K, Badell ML, Sobel J. Clinical criteria to trigger suspicion for botulism: an evidence-based tool to facilitate timely recognition of suspected cases during sporadic events and outbreaks. Clin Infect Dis 2017;66[suppl_1]:S38–S42).

**FIGURE 2:**
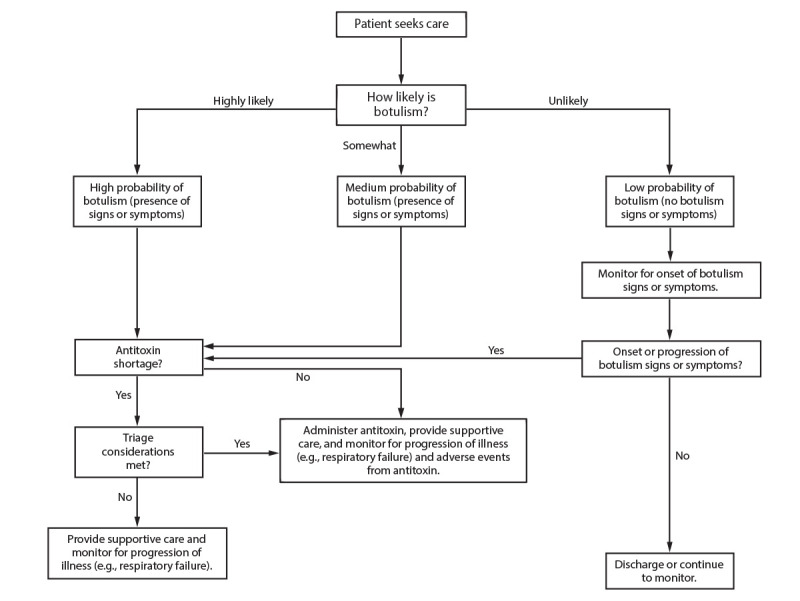
Assessing patients with known or possible exposure to botulinum toxin in crisis settings* * When assessing the likelihood of botulism, consider clinical criteria and available epidemiologic data. Classify patients into a botulism likelihood category per the clinician’s judgement. Additional information on clinical criteria is available (Rao AK, Lin NH, Griese SE, Chatham-Stephens K, Badell ML, Sobel J. Clinical criteria to trigger suspicion for botulism: an evidence-based tool to facilitate timely recognition of suspected cases during sporadic events and outbreaks. Clin Infect Dis 2017;66[suppl_1]:S38–S42).

Because diagnosing botulism can be challenging, a tool with evidence-based clinical criteria has been developed to aid clinicians in early identification of botulism in settings of crisis or contingency standards of care, when the probability of botulism increases above the level of extremely rare; the tool may be used in conventional settings as well (Box 1) (*36*). Cases of botulism from several sources were used to identify signs and symptoms of acute botulism onset, which were compared and ranked by frequency to identify criteria that are optimally sensitive for a case of botulism (Tables 3 and 4). The tool was modified to account for reasons illnesses were not captured, and expert input from clinicians and other experts was applied to the criteria that comprise the tool. Ancillary results, including those from electrodiagnostic, neuroimaging, and Tensilon (edrophonium) tests and lumbar puncture, were not included in the tool. The tool was designed for maximum objectivity and reproducibility when used among health care workers; therefore, signs that frequently occurred but were difficult to quantify (e.g., sluggishly reactive pupils) were not included. Also omitted were epidemiologic risk factors that are often not confirmed early in the course of an investigation when most severely ill patients seek care for symptoms. The tool can be used for children and adults, including pregnant women, and by various health care workers without supervision after brief, focused training during contingency and crisis situations such as large outbreaks. The tool is not intended to replace a thorough physical examination and ancillary testing or to diagnose botulism; rather, the purpose is to help clinicians determine when to consider a diagnosis of botulism, without the distractions that can result from atypical or incidental findings (*36*). In a setting of conventional standard of care, the tool can be used to stimulate consideration of botulism, followed by a more detailed evaluation. In a setting of crisis standard of care, meeting these criteria alone might be sufficient to treat for presumed botulism. If only some of the criteria are met, physicians might categorize patients as having a medium likelihood of botulism and monitor them (Figures 1 and 2). Fulfillment of these criteria should not be considered diagnostic of botulism; patients with illnesses commonly confused with botulism, including myasthenia gravis and Guillain-Barré syndrome, might meet the criteria. During outbreaks, “worried well” persons (i.e., persons who have anxiety about becoming ill during an outbreak) who do not have objective signs or symptoms of botulism often seek care at hospitals for subjective symptoms (*53*). Because triaging these patients can be time consuming and delay treatment of other patients, a response to a large botulism outbreak requires managing numerous persons who seek hospital care but do not need treatment and educating the public about which signs and symptoms do not require a hospital visit.

BOX 1 Clinical criteria tool for early diagnosis of botulism* in crisis and contingency settings^†^

- Afebrile (<100.4°F [<38°C])^§^
- Acute onset of at least one of the following symptoms:
  - Blurred vision
  - Double vision
  - Difficulty speaking, including slurred speech
  - Any change in sound of voice, including hoarseness
  - Dysphagia, pooling of secretions, or drooling
  - Thick tongue
- At least one of the following signs:
  - Ptosis
  - Extraocular palsy or fatigability (the latter manifested by inability to avert eyes from light shone repeatedly into eye [typically used in infants])
  - Facial paresis (manifested, for example, by loss of facial expression or pooling of secretions and in young children by poor feeding, poor suck on breast or pacifier, or fatigue while eating)
  - Fixed pupils
  - Descending paralysis, beginning with cranial nerves

**Source:** Rao AK, Lin NH, Griese SE, Chatham-Stephens K, Badell ML, Sobel J. Clinical criteria to trigger suspicion for botulism: an evidence-based tool to facilitate timely recognition of suspected cases during sporadic events and outbreaks. Clin Infect Dis 2017;66(suppl_1):S38–S42.

* Suspect botulism when all three criteria are met. For all patients with botulism, intact mental status is expected. If a patient has altered mental status, this might be from other causes (e.g., respiratory failure, drug or alcohol use, preexisting condition, or concurrent infection).

^†^ Although this tool can also be used in a conventional standard of care setting, a more detailed evaluation is expected. In a setting of crisis standard of care, meeting these criteria alone might be sufficient to treat for presumed botulism.

^§^ Fever concurrent with the acute onset of botulism in an adult is exceedingly rare; fever also is rare in infants and young children but might be more common than in adults.

**TABLE 3:** Signs and symptoms of patients with confirmed botulism reported in medical charts,* by frequency of signs or symptoms

Sign or symptom^†^	Frequency (%)
Afebrile^§^	99
Descending paralysis	93
Dysphagia	85
Weakness or fatigue^¶^	85
Ptosis	81
Blurred vision	80
Difficulty speaking**	78
Diplopia	75
Change in voice^††^	69
Shortness of breath^§§^	65
Dry mouth	63
Thick tongue	62
Extraocular palsy	60
Impaired gag reflex	58
Dizziness	55
Palatal weakness	54
Facial weakness^¶¶^	47
Nausea	43
Dilated pupils	37
Vomiting	33
Constipation	30
Abdominal pain	25
Abnormally reactive pupils***	24
Sensory deficits or paresthesias	17
Diarrhea	16
Urinary retention	9
Altered mental status	8
Constricted pupils	3

* N = 332. Data were obtained during clinical consultations with physicians treating botulism patients; physicians were asked about the presence or absence of signs and symptoms (**Source:** Rao AK, Lin NH, Jackson KA, Mody RK, Griffin PM. Clinical characteristics and ancillary test results among patients with botulism—United States, 2002–2015. Clin Infect Dis 2018;66[suppl_1]:S4–10).

^†^ Some symptoms of botulism are nonspecific and might resemble those of anxiety, including dry mouth, difficulty swallowing, nausea, dizziness; in settings with fewer resources, observation for more specific signs and symptoms may be considered to document progression before treatment. Ocular symptoms are considered most objective.

^§^ Defined as a temperature <100.4°F [<38°C].

^¶^ Includes complaints of generalized weakness, fatigue, or malaise.

** Includes slurred speech, trouble speaking clearly, or dysarthria.

^††^ Includes any changes in the sound of voice, such as hoarseness, nasal speech, or dysphonia.

^§§^ Includes difficulty breathing, respiratory distress, and dyspnea.

^¶¶^ Includes droop and paralysis.

*** Includes sluggish, poorly reactive, and nonreactive or fixed pupils.

**TABLE 4:** Signs and symptoms of botulism in adults with foodborne botulism, pregnant or postpartum women with botulism, and children and adolescents with any botulism syndrome, by frequency of signs or symptoms

Adults with foodborne or wound botulism* (N = 402)		Pregnant or postpartum women with botulism^†^ (N = 17)		Children and adolescents with any botulism syndrome^§^ (N = 360)	
Sign or symptom	%	Sign or symptom	%	Sign or symptom	%
Dysphagia	59	Weakness or fatigue^¶^	76	Dysphagia	52
Vomiting	42	Dry mouth	47	Dysarthria or dysphonia**	38
Diplopia	42	Shortness of breath^††^	41	Weakness or fatigue^¶^	36
Shortness of breath^††^	39	Vomiting	41	Ophthalmoplegia^§§^	32
Difficulty speaking**	39	Nausea	41	Diplopia	28
Ptosis	35	Diplopia	41	Ptosis	27
Blurry vision	33	Change in voice^¶¶^	41	Vomiting	26
Subjective weakness	32	Blurred vision	35	Shortness of breath^††^	26
Nausea	28	Dysphagia	29	Dilated pupils	26
Decreased oral secretions	25	Ophthalmoplegia^§§^	29	Abnormally reactive pupils***	22
Abnormally reactive pupils***	24	Ptosis	29	Abdominal pain	20
Ophthalmoplegia^§§^	24	Difficulty speaking**	29	Dry mouth	19
Abdominal pain	23	Descending paralysis	29	Afebrile	18
Dizziness	20	Abdominal pain	18	Constipation	17
Dilated pupils	20	Dizziness	18	Nausea	15
Change in voice^¶¶^	16	Dilated pupils	18	Dizziness	14
Fatigue	11	Facial paralysis^†††^	12	Blurred vision	14
Thick tongue	11	Poorly reactive pupils***	12	Apnea	12
Sore throat	10	Pupillary reflexes, decreased	6	Diminished gag reflex	11
Diarrhea	10	Altered mental status	6	Descending paralysis	10
Facial paralysis^†††^	8	Cranial nerve palsy unspecified	6	Sore throat	10
Neck weakness	8	Sore throat	6	Facial paralysis^†††^	10
Urinary retention	7	Areflexia	6	Urinary retention	7
Impaired gag reflex	7	Nystagmus	6	Hypotonia	6
Epigastric pain	4	Bladder distention	6	Altered mental status	3

* **Source:** Chatham-Stephens K, Fleck-Derderian S, Johnson SD, Sobel J, Rao AK, Meaney-Delman D. Clinical features of foodborne and wound botulism: a systematic review of the literature, 1932–2015. Clin Infect Dis 2018;66(suppl_1):S11–6.

^†^ **Source:** Badell ML, Rimawi BH, Rao AK, Jamieson DJ, Rasmussen S, Meaney-Delman D. Botulism during pregnancy and the postpartum period: a systematic review. Clin Infect Dis 2018;66(suppl_1):S30–7.

^§^ **Source:** Griese SE, Kisselburgh HM, Bartenfeld MT, et al. Pediatric botulism and use of equine botulinum antitoxin in children: a systematic review. Clin Infect Dis 2018;66(suppl_1):S17–29.

^¶^ Includes complaints of generalized weakness and malaise.

** Includes slurred speech, trouble speaking clearly, and dysarthria in all patients and also change in voice and weak cry in children.

^††^ Includes difficulty breathing, respiratory distress, and dyspnea.

^§§^ Includes extraocular palsy and cranial nerve 3, 4, or 6 palsies.

^¶¶^ Includes change in the sound of voice, such as hoarseness, nasal voice, or dysphonia, except in children, in whom it includes only difficulty speaking.

*** Includes sluggish, poorly reactive, nonreactive, and fixed pupils.

^†††^ Includes droop or weakness.

### Laboratory Testing

The critical initial treatment and management decisions for patients with suspected botulism must be made based on clinical findings. Botulinum antitoxin, the only specific therapy for botulism, should be administered as quickly as possible. Because laboratory confirmation can take several days, delaying administration of antitoxin to a patient with a high or medium likelihood of botulism while awaiting laboratory results can worsen the patient’s outcome (*3*,*35*,*44*,*45*).

Laboratory testing is performed to confirm clinically suspected cases, confirm that administered botulinum antitoxin contains neutralizing antibodies against the serotype of botulinum neurotoxin causing illness, and demonstrate (or confirm epidemiologic data) that botulinum neurotoxin is in the suspected food so the source can be safely removed and additional illnesses prevented. Botulism is confirmed in symptomatic persons by detecting one of the following: 1) botulinum neurotoxin in serum, stool, or gastric fluid; 2) botulinum neurotoxin–producing species of *Clostridium* (i.e., *C. botulinum*, *C. baratii*, or *C. butyricum*) in a stool or wound culture; or 3) botulinum neurotoxin in food consumed by a symptomatic person (*3*). Environmental testing is not conducted in investigations of foodborne botulism. Laboratory confirmation can only be performed by certain municipal and state public health laboratories and by CDC’s National Botulism Laboratory. Public health laboratories conduct free emergency specimen testing for possible botulism and provide detailed instructions on collection and shipment of appropriate specimens.

#### Types of Botulism Tests

The gold standard method for identifying botulinum neurotoxin, used in specialized public health laboratories, is the mouse bioassay (*54*). This method requires maintenance of mouse colonies and expertise in recognizing botulism signs in mice. Specimens are injected intraperitoneally into the mice with and without antitoxin; the mice are then observed for up to 96 hours by expert technicians for signs of botulism. Results might be available within 24 hours of receipt of the specimen by the laboratory if the botulinum neurotoxin level in the specimen is high; however, low levels of toxin that are sufficient to produce human illness might not produce signs in mice. The mouse bioassay is the only FDA-approved method for laboratory confirmation of botulism; however, other methods to detect and identify botulinum neurotoxin and botulinum neurotoxin–producing species of *Clostridium* can support a clinical diagnosis of botulism.

A real-time polymerase chain reaction (PCR) test, which is only available in reference laboratories, detects *bont* genes A–G and identifies botulinum neurotoxin–producing species of *Clostridium* in cultures. Because the PCR detects DNA and not the actual proteinaceous toxin, confirming that a strain produces toxin depends on using another method such as a mouse bioassay. The mass spectrometry method for detecting botulinum neurotoxin (Endopep-MS) is highly sensitive and specific and can differentiate among botulinum neurotoxin serotypes A, B, E, and F within several hours (*55*). This method is only available at CDC and a limited number of other public health laboratories.

Laboratory confirmation of botulism is usually not possible in nonreference laboratories (e.g., hospital and clinical laboratories) because biochemical tests and mass spectrometry performed in most of these laboratories cannot detect botulinum neurotoxin or distinguish between botulinum neurotoxin–producing *Clostridia* and nontoxigenic organisms. Occasionally, CDC is notified that a nonreference laboratory has identified *C. sporogenes* or *C. botulinum* in a clinical specimen; subsequent testing at a reference laboratory usually identifies the organism as *C. sporogenes,* which does not produce botulinum neurotoxin (CDC, unpublished data, 2018).

#### Collection and Transportation of Specimens

Serum specimens must be collected before treatment with BAT because the treatment neutralizes botulinum toxin, and subsequent testing can misleadingly indicate the absence of toxin. Laboratory confirmation of botulism depends on astute clinicians recognizing botulism signs and symptoms in patients, contacting state or local public health departments for an emergency expert clinical consultation (including discussion of laboratory testing), and ordering collection and rapid transport of appropriate specimens (Table 5; Box 2) (*2*,*3*,*28*). For laboratory confirmation of botulism, collecting clinical specimens as soon as botulism is suspected is essential to ensure that botulinum toxin, if present, is detected before it irreversibly binds within neurons and drops below the level that can be detected by the assay in serum, stool, or gastric fluid. For adults, enough whole blood should be collected without anticoagulant to yield 10–15 mL of serum (20–30 mL whole blood); a smaller volume for children is acceptable, although 4 mL of serum is the minimum volume required for the mouse bioassay.

**TABLE 5:** Specimens for botulism laboratory confirmation, by specimen type and testing parameters

Specimen type	Optimal amount	Test for botulinum toxin	Test for botulinum toxin–producing *Clostridium* species	Time from receipt of specimen by laboratory to test result*	Additional information
Serum	5–15 mL (for children: 4 mL)	Yes	No	Preliminary results for toxin in 24–48 hrs, final results in 96 hrs.	Collect before antitoxin treatment.^†^ Blood sample must be collected without anticoagulant.
Stool	10–20 g	Yes	Yes	Preliminary results for toxin in 24–48 hrs, final results in 96 hrs; final results for *Clostridium* species might take 2–3 wks.	If an enema is needed, use sterile, nonbacteriostatic water (not tap water) and non–glycerin-containing suppositories. Ideally, collect before antitoxin treatment; however, can obtain after antitoxin treatment.
Gastric aspirate	5–10 mL	Yes	Yes	Preliminary results for toxin in 24–48 hrs, final results in 96 hrs; final results for *Clostridium* species might take 2–3 wks.	Collect before antitoxin treatment.^†^
Debrided tissue, wound swab sample, or anaerobic wound culture	No specific requirements	No	Yes	Final results for *Clostridium* species might take 2–3 wks.	Broth is preferable to agar slants or plates.
Food suspected as source	10–20 g (or mL)	Yes	Yes	Preliminary results for toxin in 24–48 hrs, final results in 96 hrs; final results for *Clostridium* species might take 2–3 wks.	Ideally, the entire food item should be submitted for testing. Keep foods in original containers; if not available, place in sterile unbreakable containers. Empty containers with remnants of suspected foods can be tested.

* Times are estimates; testing might take longer during an outbreak.

^†^ Do not delay administration of antitoxin while attempting to obtain a specimen.

BOX 2 **Specimen storage and shipping**

- Specimens should be maintained at 36°F–46°F (2°C–8°C) and shipped with cold packs; do not freeze.
- Package must have proper labeling for biological hazards: UN 3373 biological substance, Category B.
- For specimens submitted to CDC for testing, follow these instructions:
  - Include a completed CDC form 50.34 in the package (available at https://www.cdc.gov/laboratory/specimen-submission/index.html).
  - On CDC form 50.34, select test order CDC-10132, Botulism Laboratory Confirmation. Include phone and fax numbers for the state health department and the hospital.
  - **Send package to:**
    STAT (Attn: Botulism Lab, Unit 26)
    Centers for Disease Control and Prevention
    1600 Clifton Rd NE, Atlanta, GA 30329
  - Contact CDC National Botulism Laboratory to provide a tracking number to CDC: https://www.cdc.gov/laboratory/specimen-submission/detail.html?CDCTestCode=CDC-10132
  - Discuss with CDC consultant whether specimens from hospitals might need to be submitted through the local or state health department or state public health laboratory.

Although 10–20 g of stool should be collected, smaller amounts are sometimes sufficient; rectal swabs from infants or young children are acceptable. In constipated patients, stool specimens can be collected by performing an enema with (preferably) sterile nonbacteriostatic water and non–glycerin-containing suppositories; tap water can interfere with laboratory testing and is not recommended. Stool may be collected after treatment with BAT because *Clostridium* organisms might still be present in stool even if toxin has been neutralized in the serum (*17*); BAT treatment should not be delayed to collect stool specimens. Suspect foods should be sent for laboratory testing in their original containers so laboratory experts can determine which parts of the food specimen should be tested. Even containers with dried or sparse amounts of food have yielded positive test results (CDC, unpublished data, 2016). If necessary, food can be sent in sterile, unbreakable containers. All clinical and food specimens should be immediately refrigerated (36°F–46°F [2°C–8°C]) and kept at this temperature while transported; specimens should not be frozen. Specimens from exposed but asymptomatic persons are not routinely tested because the toxin in their specimens is likely to be below the limit of detection of the mouse bioassay; rare exceptions include known exposures to high toxin levels in a research laboratory setting, in which clinical specimens can be obtained and BAT can be administered before illness onset.

Confirmation of botulism in a sporadic (single) patient is valuable because it eliminates alternative diagnoses and their treatments and provides a prognosis. In a substantial proportion of cases, test results are negative despite near certainty of the clinical diagnosis. This typically occurs because a delay in recognizing possible botulism leads to collection of clinical specimens later in illness, when levels of toxin in serum have fallen below the limit of detection of laboratory tests. In patients with wound botulism, botulinum neurotoxin–producing species of *Clostridium* are not always detected in wound specimens, especially after administration of antibiotics.

#### Recommendations

- Treat patients with suspected, symptomatic botulism with botulinum antitoxin on the basis of clinical findings; do not await laboratory confirmation because results might take several days, and they can be negative in patients who have botulism. (For risks and benefits of BAT treatment, see Allergic Reactions and Other Side Effects of Botulinum Antitoxin.)
- Discuss specimen collection with the expert consultant from CDC or the local or state health department.
- Collect specimens for laboratory confirmation of the clinical diagnosis of botulism as soon as possible because toxin levels decrease over time (Table 5; Box 2). Obtain serum before BAT is administered.
- Store and transport specimens for botulism testing at refrigeration temperatures (36°F–46°F [2°C–8°C]); do not freeze.

## Monitoring Illness Progression in Patients with Botulism

Botulism causes progressive flaccid, descending paralysis that might result in respiratory compromise from upper airway collapse or respiratory muscle impairment (*3*). Patients with botulism should be monitored closely for neurologic, respiratory, and autonomic manifestations. Because of the potential for rapid clinical deterioration, frequent examination and other monitoring measures should be performed to allow prompt life-saving interventions.

### Neurologic Monitoring

Botulism signs and symptoms occur in a typical order. Some patients initially have nausea and vomiting, then nearly all patients develop cranial nerve palsies (which might include respiratory compromise from upper airway compromise); some develop respiratory failure and paralysis of the extremities (*14*). A systematic review of 375 patients in the literature documented a range in the number of cranial nerve palsies recorded at hospital admission: 126 (34%) patients had one or two cranial nerve palsies, 119 (32%) patients had three or four, and 130 (35%) patients had five or more; 27 (7%) had no cranial nerve palsies noted (*14*). Paralysis can progress rapidly (*14*).

### Respiratory Monitoring

Neuromuscular paralysis in patients with botulism can result in respiratory failure by affecting the muscles that prevent aspiration and maintain a patent upper airway, as well as those involved in respiration (e.g., the diaphragm). In a systematic review of 402 adults with botulism, 169 (42%) patients had respiratory compromise when admitted to the hospital, with either shortness of breath or dyspnea (50 [12%]) or respiratory distress or failure (119 [30%]) (*14*). Almost half (184 [46%]) later required intubation and mechanical ventilation. Approximately two thirds of patients who had respiratory involvement when admitted to the hospital had been ill for <48 hours. Among patients who required intubation (with data on hospital day of intubation), 87% required intubation in the first 2 hospital days. In a review of medical charts from 99 patients with confirmed botulism, shortness of breath was reported in 67 (68%) patients on the first or second day of hospitalization (CDC, unpublished data, 2016). In a systematic review of 17 pregnant patients with botulism, 11 (69%) patients experienced respiratory failure requiring intubation and mechanical ventilation (*30*). Pregnant patients might be at increased risk for respiratory failure because of decreased functional residual lung capacity, diaphragmatic rise, increased oxygen consumption, and increased intra-abdominal pressure (*56*). In a systematic review of 360 children with botulism, 91 (25%) required mechanical ventilation (*31*). Adult, nonpregnant patients with botulism might require mechanical ventilation more frequently than children with botulism because of comorbid conditions such as chronic obstructive pulmonary disease and obesity.

No data are available to indicate whether certain clinical features among patients with botulism are associated with eventual intubation and mechanical ventilation. An analysis of 20 patients with wound botulism found no statistically significant differences in the initial signs and symptoms of patients who developed respiratory failure and patients who did not (*57*). Nausea, vomiting, and any cranial nerve palsy with urinary retention or dysphagia were the signs and symptoms most predictive of respiratory failure in an analysis of 137 patients from a foodborne botulism outbreak in Thailand (*58*).

Because of the paucity of data regarding respiratory monitoring and clinical predictors of respiratory failure in patients with botulism, clinical practices from other neuromuscular disorders that can cause respiratory failure (e.g., myasthenia gravis and Guillain-Barré syndrome) might be used for respiratory monitoring of patients with botulism. Spirometry is an objective measure of respiratory muscle function that, along with physical examination, can help clinicians determine whether a patient needs intubation. For example, forced vital capacity (FVC) <20 mL/kg, maximum inspiratory pressure (i.e., negative inspiratory force) <30 cm H_2_O, and maximum expiratory pressure <40 cm H_2_O were each associated with the need for mechanical ventilation in Guillain-Barré syndrome patients (*59*).

Respiratory function also might be monitored using sniff nasal inspiratory pressure and the single breath count test. Sniff nasal inspiratory pressure testing, which evaluates diaphragm strength and inspiratory muscle function, involves occluding one nostril with a pressure-measuring device and inhaling sharply through the other nostril (*60*,*61*).Values greater than −70 cm H_2_O (males) or −60 cm H_2_O (females) might reflect the absence of clinically significant inspiratory muscle weakness; severe nasal congestion might cause falsely low values (*62*,*63*).The single breath count test involves taking a deep breath and then counting at a rate of two numbers per second for as long as possible while exhaling. A study of 31 patients with myasthenia gravis documented that the single breath count correlated with FVC, with each counted number equal to 116 mL of FVC; counting to ≥25 was proposed to correlate with normal respiratory muscle function (*64*). End-tidal carbon dioxide (EtCO_2_) monitoring, which is noninvasive and available in many hospitals, is an optional modality for monitoring early respiratory failure. Rising partial pressure of CO_2_ (pCO_2_) or EtCO_2_ strongly predicts the need for mechanical ventilation. Bulbar dysfunction is often a prominent feature of botulism and has been associated with the need for intubation and mechanical ventilation in patients with Guillain-Barré syndrome (*59*). Monitoring pulse oximetry and arterial blood gases might not be reliable early indicators of emerging respiratory failure in patients with botulism because hypoxia and hypercapnia might not develop until the later stages of respiratory failure, as documented in patients with other neuromuscular disorders in which gas diffusion is unimpaired, such as Guillain-Barré syndrome (*65*–*67*).

### Autonomic Function Monitoring

Botulinum neurotoxins can impair the postganglionic release of acetylcholine in the parasympathetic nervous system, causing unopposed stimulation of the sympathetic system (*68*,*69*). Case reports and series have noted features of dysautonomia (e.g., dry mouth, urinary retention, constipation, and orthostatic hypotension) in patients with botulism, usually in cases caused by toxin type B (*70*–*73*). Similarly, autonomic dysfunction is commonly reported in Guillain-Barré syndrome patients, with cardiac manifestations of dysautonomia, including sinus tachycardia or bradycardia, cardiac dysrhythmias, blood pressure lability, abnormal hemodynamic responses to medications, and nonspecific electrocardiogram changes (*74*–*76*). These manifestations have prompted some experts to recommend close monitoring of pulse and blood pressure in patients with Guillain-Barré syndrome (*77*). Similar monitoring would therefore be reasonable for patients with botulism.

### Recommendations

- Conduct frequent, serial neurologic examinations, with an emphasis on cranial nerve palsies, swallowing ability, respiratory status, and extremity strength.
- In settings of contingency and crisis standards of care, in which time is limited, focus examinations on signs and symptoms of early onset (Box 1). Consider brief, focused training in the emergency setting on the neurologic examination.
- When possible, have the same health care provider conduct the serial neurologic and other examinations.
- Adjust the frequency of neurologic and other examinations on the basis of signs and symptoms, with very frequent examinations for patients with rapid progression and for patients who have respiratory or bulbar symptoms but have not required intubation.
- Institute frequent, serial monitoring of respiratory and bulbar function. Serial measurements might be more helpful than a single measurement.
  - Focus the respiratory examination on respiratory rate, lung field auscultation, and work of breathing, including use of accessory muscles of respiration, nasal flaring, and paradoxical breathing (*78*).
    1. Obtain serial objective data through spirometry, EtCO_2_ monitoring, blood gas analysis, or other tests. Patients with facial weakness might not achieve an adequate seal around the spirometer mouthpiece and so might require a mask device (*78*,*79*). If spirometry is not available, consider using the sniff nasal inspiratory pressure or the single breath count test.
    2. Consider respiratory status in the context of neurologic status because paralysis can alter signs typically associated with respiratory distress. For example, facial paralysis can produce a placid expression that can obscure distress from respiratory insufficiency and also prevent nasal flaring, and diaphragmatic paralysis can result in paradoxical abdominal movement in which the abdomen moves inward during inspiration (*80*).
  - Focus bulbar dysfunction examination on dysphagia, dysarthria, nasal voice, drooling, and impaired gag reflex (*78*). When feasible, consider assessing the patient’s swallowing ability to help determine whether the patient can safely consume liquids or solids (*81*).
- Continuously monitor cardiac rhythm and frequently measure blood pressure.
- Frequently monitor for urinary retention, constipation or ileus, dry mouth, and dry eyes.

## Treatment Considerations

Treatment involves supportive care, intubation and mechanical ventilation when necessary, and administration of equine-derived botulinum antitoxin. Botulism produces a protracted flaccid paralysis that lasts for weeks to months. Death in the acute state is typically the result of early respiratory failure; later in the course of illness, death is usually caused by complications from protracted intensive care, such as ventilator-associated pneumonia and deep vein thrombosis (DVT) (*3*). Timely administration of botulinum antitoxin mitigates the extent and severity of paralysis, including, in certain instances, prevention of progression to respiratory compromise, and in other instances, reduction of the duration of mechanical ventilation and intensive care (*31*,*37*,*82*,*83*).

Almost all patients with botulism can survive, even without antitoxin, if they receive supportive care, including mechanical ventilation, when required. This is reflected in the mortality trend for botulism. The case-fatality ratio approached 70% in the first half of the twentieth century, despite the availability of botulinum antitoxin then (*82*). Mortality rates decreased beginning in the 1940s and 1950s to their current rate of <5%, with improvement corresponding to the development of modern intensive care techniques, particularly mechanical ventilation (*82*). However, survival and recovery require prolonged use of intensive care resources, which might be limited in events with many patients with botulism.

## Botulinum Antitoxin Treatment

### Background

The only specific therapy for botulism is botulinum antitoxin. When administered early in the course of illness (within 48 hours of symptom onset and ideally within 24 hours), botulinum antitoxin can stop the progression of paralysis and prevent respiratory compromise in certain patients. The antitoxin cannot reverse existing paralysis. The antitoxin is an equine-derived preparation of antibodies that bind and neutralize botulinum toxin in the bloodstream that has not yet irreversibly bound to synaptic receptors; the resulting antitoxin-toxin complex is cleared from circulation (*29*,*37*). Botulinum antitoxin is toxin type–specific (e.g., antitoxin to toxin type A neutralizes only toxin type A). BAT, the only botulinum antitoxin preparation available for treatment of noninfant botulism in the United States, is a mixture of antibodies to botulinum toxin types A, B, C, D, E, F, and G, licensed for the treatment of symptomatic botulism in adults and children (*29*,*37*,*84*). Antitoxin is stocked by CDC, and health care providers who identify an illness they suspect is botulism can contact the 24-hour CDC botulism consult service and request antitoxin if indicated (https://www.cdc.gov/botulism/health-professional.html).

### Recommendation

- Health care providers who suspect botulism on the basis of clinical symptoms should immediately call the emergency contact number of their local or state health department to arrange for an emergency clinical consultation and, when indicated, shipment of antitoxin (https://www.cdc.gov/botulism/health-professional.html).

### Dose

The standard adult dose* is one vial, administered by intravenous infusion. The pediatric dose is based on weight.

The standard adult dose of BAT contains approximately 10^7^ IU of antitoxins A, B, C, and F; 10^6^ IU of antitoxins D and E; and 600 units of antitoxin G (Table 6). These amounts exceed by one to two orders of magnitude (i.e., tenfold to 100-fold greater) the amount of toxin types A, B, or E documented in the serum of virtually every botulism patient in whom the toxin level has been quantified. A theoretical possibility exists that the neutralizing capacity of BAT could be exceeded by circulating toxin levels in a patient exposed to an extremely high toxin load in naturally occurring disease; the circulating toxin level that might be attained in an intentional contamination event is not known.

**TABLE 6:** International units of antitoxin and neutralization capacity in one vial of botulinum antitoxin, by serotype*

Serotype	Antitoxin	Neutralization capacity
	IU per vial	MIPLD_50_ per vial
A	4,500	4.5 × 10^7^
B	3,300	3.3 × 10^7^
C	3,000	3.0 × 10^7^
D	600	6 × 10^6^
E	5,100	5.1 × 10^6^
F	3,000	3.0 × 10^7^
G	—^†^	6 × 10^6^

**Abbreviations:** IU = international units; MIPLD_50_ = mouse intraperitoneal lethal dose_50_.

* The standard adult dose is one vial (**Source:** Botulism antitoxin heptavalent [A, B, C, D, E, F, G—equine] [package insert]. Gaithersburg, MD: Cangene Corporation [Emergent Biosolutions]; 2017. https://www.fda.gov/media/85514/download).

^†^ Six hundred units that are not recognized internationally.

### Allergic Reactions and Other Side Effects of Botulinum Antitoxin

#### Background

BAT contains purified antibodies from the serum of horses immunized with botulinum toxoids and toxins. As concentrated preparations of foreign proteins, they can elicit immune reactions, including anaphylaxis, in human recipients. Despeciation and other processes used in producing modern equine antitoxin might reduce but do not eliminate the risk for allergic reactions. Data on BAT indicate an anaphylaxis rate of <2%; a similar frequency was calculated for previously used formulations (*29*,*31*,*37*,*85*). Among 249 patients treated with BAT in one analysis, the only serious adverse event occurred in a child who experienced hemodynamic instability, including asystole, and recovered; other allergic reactions, typically rash, were noted in six patients and were without sequelae (*37*). Skin testing before antitoxin administration, once universally recommended, is no longer recommended. Skin testing requires specialized training, is cumbersome and time consuming, and is likely to have a low positive predictive value (*29*,*37*). Serum sickness has been reported among antitoxin recipients; the frequency is not well established (*37*).

#### Recommendations

- Do not routinely perform skin testing for sensitivity before BAT administration.
- Ensure that epinephrine and antihistamine treatments are available for all patients receiving BAT. Caregivers capable of identifying and responding to anaphylaxis should observe patients during antitoxin administration.

### Guiding Principles of Antitoxin Treatment

Administration of botulinum antitoxin early in the course of illness decreases mortality, duration of treatment in the intensive care unit, and duration of hospitalization (*27*,*31*,*37*,*82*,*83*). A systematic review and meta-analysis of data during 1923–2016 indicated that overall, antitoxin reduced mortality (odds ratio [OR] = 0.22; 95% confidence interval [CI] = 0.17–0.29), with the greatest reduction associated with treatment of botulism type E (OR = 0.13; 95% CI = 0.06–0.30), followed by botulism type A (OR = 0.57; 95% CI = 0.39–0.84). Reduction in mortality was not statistically significant for type B botulism (OR = 0.74; 95% CI = 0.27–1.97), possibly because this toxin type causes milder disease. These findings are driven by data from patients treated with anti-ABE trivalent antitoxin, which, when administered for botulism types A, B or E, significantly reduced overall mortality (OR = 0.13; 95% CI = 0.04–0.38) (*82*).

A systematic review of botulism in children found that antitoxin administration (multiple formulations over many decades) significantly reduced mortality (relative risk [RR] = 0.65; 95% CI = 0.53–0.80; p<0.001). When evaluated by age group, patients aged 1 to <5 years (RR = 0.43; 95% CI = 0.20–0.93; p = 0.007) and aged 5 to <9 years (RR = 0.52; 95% CI = 0.33–0.82; p<0.001) who received antitoxin had significantly decreased relative risk for death. The relative risk of the remaining age groups followed a similar trend (*31*). Although a systematic review of 17 cases of botulism in pregnant women did not find a significant association between antitoxin administration or early antitoxin administration and improved outcome, the trend observed suggested such a relation (*30*).

### Timing of Botulinum Antitoxin Administration

#### Administration of Antitoxin Early in the Course of Illness

Among 104 patients with confirmed botulism treated with BAT, those treated within 2 days after illness onset spent fewer days in the hospital (median: 15 versus 25 days; p<0.01) and in the intensive care unit (10 versus 17 days; p = 0.04) than those treated later (*37*). A systematic review and meta-analysis reported similar findings from reports published over nearly a century and entailing the use of various antitoxin formulations (*82*). One study from 1984 reported that among 132 patients with type A botulism, those who had received trivalent anti-ABE equine antitoxin had a lower fatality rate and a shorter course of illness than those who did not receive antitoxin, controlling for age and incubation period. Patients who received antitoxin within 24 hours after symptom onset had a shorter course but similar fatality rate as those who received antitoxin later (*83*). Another study from 2006 reported on a subset of 18 severely ill patients from a large botulism type A outbreak in Thailand. In this subset, patients who received antitoxin on day 4 of illness onset had significantly shorter duration of ventilator dependence than those receiving it on day 6 (*86*).

#### Recommendation

- Administer botulinum antitoxin to patients with suspected botulism as early as possible in the course of illness. The greatest benefit accrues to those who receive it within the first 2 days of illness onset.

#### Administration of Antitoxin Later in the Course of Illness

Neutralizing any circulating toxin should be beneficial. Available evidence does not indicate a point in the course of illness beyond which antitoxin administration provides no benefit (*82*). One study found that for 309 foodborne botulism cases, toxin was detected in approximately 20% of serum specimens collected beyond the sixth day of illness (*17*). However, toxin was detected in the circulation of one foodborne botulism patient 12 days after and in another 25 days after symptom onset (*44*). Because paralysis that continues to progress indicates that toxin is still circulating, such patients should receive antitoxin to protect unaffected muscles, regardless of the number of days after illness onset. Because current tests to identify toxin in patient serum take several days, treatment decisions should not be delayed in anticipation of test results. Antitoxin does not reverse paralysis. Recovery from paralysis takes weeks to months, even after antitoxin administration.

#### Recommendations

- Patients with suspected botulism whose symptoms or signs (e.g., paralysis) are progressing should be treated with BAT regardless of the time that has elapsed since symptom onset.
- Patients with suspected botulism whose symptoms and signs are not progressing and who have no remaining voluntary muscle function are less likely to benefit from antitoxin treatment, especially if >7 days have passed since symptom onset, because toxin is infrequently detected beyond this point of illness.

### Characteristics of Patients and Success of Antitoxin Administration

#### Background

No available evidence indicates that any particular patient characteristic (e.g., age, sex, or preexisting health conditions) predicts better outcome from antitoxin administration (*82*). One report found that among 132 patients with botulism type A, the reduction in fatality rate and duration of illness associated with antitoxin administration persisted after controlling for age (*83*). General principles of respiratory care suggest that patients with preexisting respiratory conditions (e.g., obstructive or restrictive lung disease) or physiologic or anatomic conditions (e.g., pregnancy, obesity, and chest wall malformation) might have a higher risk for respiratory compromise than the general population. Earlier administration of antitoxin to such patients theoretically might have more impact in preventing respiratory failure.

#### Recommendation

- Patients with suspected botulism should be treated with BAT regardless of underlying medical conditions or age, sex, or other demographic characteristics.

### Retreatment of Adults

#### Background

Retreatment for a single exposure to botulinum toxin, which would imply circulating toxin levels exceeding the antitoxin’s neutralizing capacity, is not described in the modern published literature. Toxin circulation has been reported in untreated patients 12 days and 25 days after exposure (*44*). Such persistent presence of toxin from a single exposure suggests an extremely high exposure dose and initial circulating level, very slow absorption of ingested toxin, or development of a botulinum toxin–producing colony of *C. botulinum* in the patient’s intestine after ingestion of a food contaminated both with spores and toxin. Cases such as this are exceedingly rare.

Antitoxin prevents progression of paralysis; antitoxin administration is not followed immediately by reversal of paralysis already present at the time of administration. In an outbreak or another situation in which the clinical diagnosis of botulism is certain, progressive neurologic illness >24 hours after treatment suggests a circulating toxin level exceeding the neutralization capacity of administered antitoxin. In a person with a suspected sporadic (single) case, especially with atypical features or other circumstances associated with less diagnostic confidence, progression of paralysis despite antitoxin treatment should increase consideration of alternative diagnoses. Under unusual circumstances, such as documented exposure to high levels of toxin (e.g., unusually high toxin content in food or atypically high toxin levels in patients’ circulation), public health officials could recommend increasing or repeating the antitoxin treatment. However, because toxin quantification is not routinely performed, such information is unlikely to be available.

The half-life (t_1/2_) in patients’ circulation of the seven antitoxin types in one vial ranges from 7.5 to 34 hours (*84*). Theoretically, a shorter half-life might result in reduced neutralization of toxin that is being absorbed from the gut into the circulation over time. In the highly rare instance in which it is clinically indicated, a second dose of BAT given within 2 weeks is unlikely to result in a hypersensitivity reaction related to sensitization caused by the first dose because it usually takes longer for the immune system to respond to a new antigen (*29*). Older formulations of botulinum antitoxins had longer half-lives than BAT (*84*,*87*).

#### Recommendations

- Do not give patients with suspected botulism a second dose of BAT unless progression of paralysis clearly continues after the initial dose should have taken effect and suspicion for botulism is high.
- If neurologic signs progress for >1 day after administration of one vial of BAT, consider diagnoses other than botulism.

### Infants and Children

#### Infant Botulism Syndrome

The syndrome known as infant botulism is an exceedingly rare and sporadic disease caused by colonization of the intestine by Clostridia and in situ toxin production. A single case of suspected botulism in an infant is usually presumed to be infant botulism. These guidelines do not address the syndrome of infant botulism, for which the indicated treatment is human-origin anti-A, anti-B botulinum antitoxin (BabyBIG), available after consultation from the California Department of Public Health Infant Botulism Treatment and Prevention Program. Infant botulism syndrome caused by other toxin types may be treated with BAT (*88*). However, infants can be affected by toxin that they ingest. If an infant is affected as part of a group of botulism cases, the infant has likely been exposed to a toxin from food or the environment, and the illness is likely to be botulism in an infant rather than the syndrome of infant botulism. In such circumstances, the infant should receive BAT and be treated using these guidelines.

#### Dose

The FDA-approved BAT dose for infants (persons aged <1 year) is 10% of the adult dose, regardless of weight. The BAT dose for children (persons aged 1–16 years) is 20%–100% of the adult dose, according to 10 weight-based categories (*84*). These doses were derived using the Salisbury rule, which is a method of calculating weight-based brackets for dosing in children (*89*): for children who weigh <66 lb (30 kg), double the body weight to determine the percentage of the adult dose to use; for children who weigh >66 lb (30 kg), add 66 lb (30 kg) to the body weight to determine the percentage of the adult dose to use. However, weight-based dosing might not provide a dose of sufficient neutralizing capacity in a child.

Botulinum antitoxin acts by neutralizing toxin in the circulation not yet bound to synaptic nerve endings. Therefore, the amount of toxin in circulation is not proportional to the patient’s weight and instead reflects the dose of toxin ingested. The dose ingested might not be proportional to the volume of food consumed because the distribution of toxin in a food can vary widely. The absolute amount of botulinum toxin in a child might be no different from, but could also be greater than, the amount in an adult who ate the same contaminated food. Therefore, the amount of neutralizing antitoxin required needs to be proportional to the amount of toxin present and could result in the same (or greater) dose than for an adult.

Treatment of pediatric botulism without regard to body weight has been reported in one publication, which documented that 12 patients aged 4–61 years in Sichuan Province, China, all received 100,000 units of antitoxin, with no adjustment of dose for age or weight. Although no data were reported on severity of morbidity or outcome by age, the length of stay in the hospital ranged from 5 to 19 days (median: 8 days), with no deaths (*90*). Several other case reports involving children have been published but without specific data on dosing regimen.

As noted in the sections pertaining to botulinum antitoxin treatment of adults, if a child needs a second dose of BAT (a situation that is highly unusual and is clinically indicated by progression of paralysis >24 hours after administration of a first dose of antitoxin, with high confidence in the diagnosis of botulism), the dose is unlikely to result in a hypersensitivity reaction because of sensitization caused by the first dose. The immune system takes weeks to complete the humoral immune response after introduction of antigen; therefore, an allergic reaction to a second dose within a period of up to 2 weeks would be unlikely (*29*).

#### Key Point for Clinicians

- Although weight-based dosing of BAT is advised in the package insert, evidence is lacking to suggest this method is more effective than dosing based on toxin load or that adverse reactions are dose related. Children who have ingested a large amount of toxin might require more antitoxin than is indicated by the weight-based dose described in the BAT package insert.

#### Recommendation

- Children suspected of having foodborne botulism and treated with BAT according to the weight-based dose described in the package insert should be monitored closely for worsening paralysis. When confidence in the diagnosis of botulism is substantial, a lack of response to the treatment might indicate that the dose was insufficient, and retreatment should be considered.

### Pregnant Women

#### Background

A systematic review assessed 17 cases of botulism among pregnant women treated with antitoxin. No antitoxin-related complications were reported in the patients or their fetuses. Although statistical testing of the therapeutic effects of antitoxin administration was not performed because of the small number of cases, descriptive findings suggested that, as in nonpregnant patients, antitoxin improved outcomes when administered early (*30*).

General principles of respiratory care suggest that preexisting respiratory diseases (e.g., obstructive or restrictive lung disease) or physiologic or anatomic conditions (e.g., pregnancy, obesity, and chest wall malformation) might increase the risk for respiratory compromise in patients with botulism compared with those in the general population. Earlier antitoxin administration might help prevent respiratory failure in these patients.

#### Recommendation

- Pregnant women with suspected foodborne botulism should be treated with BAT in the same manner as nonpregnant patients.

### Antitoxin Shortages

#### Background

The U.S. government has a large stock of BAT. Vials are stored in facilities of the Strategic National Stockpile in readiness for immediate shipment (*3*). A temporary shortage might occur at a clinical facility treating patients because of the time required for shipment and distribution. Theoretically, a shortage could occur during a very large outbreak in which cases exceed the doses in the national stockpile. Depending on the severity of shortage, contingency or crisis standards of care might apply to the antitoxin supply.

Under conditions without resource restrictions, BAT is typically administered to patients with progressing signs of botulism; the risks from BAT administration in a carefully monitored setting with readily available intensive care are quite low, and the benefits of preventing progression of paralysis are substantial (*29*,*37*). Antitoxin should not be administered to asymptomatic patients. In settings requiring contingency or crisis standards of care because of a BAT shortage, increasingly restrictive criteria are applied to identify the patients more likely to benefit from antitoxin administration. Available data do not provide unequivocal clinical characteristics or features that identify patients more likely to benefit from antitoxin treatment. However, certain considerations might assist physicians in making antitoxin administration decisions during an antitoxin shortage.

The overarching objective of antitoxin treatment is to prevent respiratory failure, the principal cause of death in the early stages of botulism. Treating respiratory failure requires intubation and mechanical ventilation, with its attendant risks and complications, as well as resources for protracted intensive care and hospitalization. These steps might in turn create or exacerbate shortages of ventilators and other resources. Thus, when there is a shortage of ventilators, antitoxin should be used in a way that minimizes the occurrence of respiratory failure (i.e., administration of antitoxin to patients who do not yet require intubation but whose illness might still be progressing).

Early administration of botulinum antitoxin (≤2 days from symptom onset) reduces overall death and duration of hospitalization. Among patients who have not experienced respiratory failure, data are insufficient to indicate with certainty those who will benefit most from antitoxin treatment. Some patients with mild symptoms will not progress to experiencing respiratory compromise even without treatment, and some patients with rapidly progressing botulism, even when treated with antitoxin on the first day of symptoms, nevertheless require intubation and mechanical ventilation (*38*).

Staff should conduct frequent, focused neurologic examinations of patients who do not need intubation to identify those whose signs and symptoms are progressing and who are at greater risk for respiratory failure (see Monitoring Illness Progression in Patients with Botulism). Patients whose signs and symptoms progress rapidly (over hours) are likely at highest risk. However, factors associated with respiratory failure among patients who received prompt treatment with antitoxin are not known, nor is which patients would have required intubation had they not received antitoxin. Also unknown is whether antitoxin administered to patients whose neurologic findings are progressing decreases the duration of mechanical ventilation.

The standard adult dose of BAT (one vial) contains sufficient amounts of antitoxin types A, B, C, E, and F to neutralize approximately 100-fold the measured serum toxin level for virtually all patients with botulism type A, B, or E in whom circulating botulinum toxin levels have been quantified and sufficient antitoxin of types D and G to neutralize approximately tenfold the measured serum toxin level. These figures suggest that the standard adult dose of BAT could reasonably be divided among two or more patients during a shortage while maintaining an acceptable excess of neutralization capacity. However, there is no known limit to the dose of botulinum toxin that can be ingested, nor is there a known maximum serum level that can be attained in persons who have botulism from unintentional exposure or from a deliberate contamination event.

Ethical decision-making on the allocation of scarce resources during a shortage is not limited to using resources in the manner that is most clinically appropriate. This type of decision-making requires incorporating principles of fairness and equity, meaning that all persons equally likely to benefit from therapy have a similar chance of receiving treatment. Allocation schemes should take into account community preferences. For example, in some communities or cultures, children are considered a privileged, or priority, group. During a shortage emergency, new procedures that incorporate clinical criteria, standards of fairness, equity, and community preferences for allocation of antitoxin cannot be satisfactorily established. Allocation approaches and criteria should be developed as part of emergency planning, using a deliberate, transparent process that incorporates the full range of stakeholders, including those who can articulate the preferences of the community.

#### Key Points for Clinicians

- Carefully assessing history of illness and monitoring patients to identify those at greatest risk for progress to respiratory arrest might help in decision-making.
- Patients who do not require intubation but have progressing signs and symptoms are at highest risk for developing respiratory compromise.
- Patients who do not require intubation, do not have respiratory compromise, and are reliably observed to have stable (nonprogressing) signs or symptoms might be considered to be at less risk for developing respiratory compromise.
- Patients who do not require intubation but have progressing signs or symptoms are likely to receive some benefit from antitoxin administration.
- Limited data indicate that patients who seek care >7 days after illness onset are less likely to have botulinum toxin in circulation.
- No demographic criteria can be used to definitely identify patients with botulism who are more likely to benefit from antitoxin treatment.

#### Recommendation

- Proactively develop an approach to handle BAT shortages as part of an emergency planning process that incorporates the full range of stakeholders, including local communities.

## Treatments Other than Botulinum Antitoxin

A meta-analysis found no evidence for effectiveness of any specific treatment other than botulinum antitoxin to date (*82*). Research is ongoing for certain modalities addressed in this section.

### Activated Charcoal, Polyethylene Glycol, Cholinergic Agonists, and Plasmapheresis

No data exist on the effectiveness of activated charcoal in the treatment of humans with botulism. A study in mice showed that adding activated charcoal to botulinum toxin injected intraperitoneally provided full protection from signs and death (*91*). However, oral administration of activated charcoal in humans might be associated with complications. For example, aspiration and resultant pneumonitis could occur among patients with decreased gag or swallow reflexes (*92*), and activated charcoal in the gut might complicate the management of ileus. Although polyethylene glycol preparations have been proposed to speed efflux of toxin from the gut, no evidence of benefit exists (*93*). Cholinergic agonists such as guanidine and 3,4-diaminopuridine have been used in attempts to stimulate acetylcholine release because they have been used in the treatment of other neuromuscular illnesses, with apparently transient effects (*94*–*101*). Patients have also been treated with plasmapheresis, with no clear benefit (*102*).

### Antimicrobials

Antimicrobials do not provide any benefit in treatment of botulism (*82*). Theoretical concerns have been raised concerning increased botulinum toxin release from lysed Clostridia organisms after antimicrobial treatment (*103*). Wound botulism is caused by clostridial colonization of an anaerobic wound, treatment of which is generally centered on debridement (*3*); treatment should address each patient’s clinical situation.

Aminoglycosides act in vitro as neuromuscular blocking agents (NMBAs) and aggravate botulism anecdotally in several animal species and in humans with infant botulism (*104*–*106*). In addition, aminoglycosides have been reported as an aid for diagnosing botulism in mouse models (*104*). The neuromuscular blocking potency is highest with neomycin and decreases sequentially with gentamicin, streptomycin, kanamycin, amikacin, and tobramycin (*107*). The effect is more likely to occur with serosal administration (e.g., intraperitoneal) but has been reported by all routes and with a higher incidence in patients also receiving anesthetics, NMBAs, or both, and with massive transfusions of citrated blood (*108*). The suggested mechanism of action is a reduction in presynaptic calcium uptake and acetylcholine release similar to that caused by magnesium, as well as postsynaptic binding. This has been postulated to be reversible with calcium salts, although possible calcium toxicity from such treatment is a concern (*108*).

### Medications that Should Be Used with Caution

Most of the following agents are thought to pose theoretical risks to patients with botulism. (Substantive evidence exists for the risk associated with aminoglycosides.)

#### Antimicrobials

Concerns exist about the ability of clindamycin to block acetylcholine release, and its action might work together with that of aminoglycosides (*103*,*109*). Theoretical concerns exist regarding penicillins increasing toxin load through cell lysis and with tetracycline through chelation of calcium (*109*). However, avoiding the use of these agents in a patient with a comorbid infection must be weighed against the benefits of treating the comorbid condition. Patients with botulism who are being treated with antimicrobial agents should be observed for clinical deterioration that could be related to receiving the antibiotic.

#### Magnesium, Calcium, and Monoamine Oxidase Inhibitors 

Magnesium, a competitive inhibitor of presynaptic calcium-dependent acetylcholine release, produces dose-dependent skeletal muscle paralysis and prolongs paralysis from NMBAs and diseases such as myasthenia gravis (*110*). In a cattle study, calcium infusions were reported to increase paralysis and dissemination of botulinum toxin (*111*). Calcium-channel blockers (e.g., verapamil, nifedipine, and diltiazem) can interact with aminoglycosides to produce complete neuromuscular blockade among patients who do not have botulism and theoretically should be avoided on the basis of this interaction (*112*,*113*). A single report has been published of pretreatment of mice with the monoamine oxidase inhibitor pargyline resulting in rapid botulism-induced death (*114*).

#### Neuromuscular Blocking Agents

Any agent that can cause paralysis, including NMBAs, should either be avoided or be used after careful consideration and with appropriate monitoring. The NMBA succinylcholine induces sustained depolarization of motor endplate at the myoneuronal junction. The nondepolarizing NMBAs, such as rocuronium, vecuronium, and pancuronium, block acetylcholine from binding to motor endplate receptors.

### Key Points for Clinicians

- Patients with suspected, symptomatic botulism should be treated with BAT and receive supportive care (e.g., intensive care including intubation and mechanical ventilation when necessary).
- Evidence does not indicate benefit from any treatment modalities other than antitoxin, although data are limited.

### Recommendation

- Aminoglycosides, magnesium, clindamycin, tetracycline, or calcium should only be administered to patients with botulism after careful consideration and with appropriate monitoring.

## Botulism, Antitoxin, and Breast Milk

### Background

Three botulism cases have been reported in breastfeeding women, prompting questions about whether such breast milk can be safely fed to infants. Whether botulinum toxin enters breast milk is not known; this issue has not been systematically researched. Many factors influence whether a compound is transferred from serum to breast milk (e.g., molecular weight and lipid solubility) (*115*). Medications with a molecular weight >800 daltons are less likely to achieve clinically relevant levels in breast milk than smaller compounds (*115*). The molecular weight of botulinum toxin (150,000 daltons) might prevent its passage into breast milk (*116*).

The three breastfeeding women who breastfed their children while ill with botulism are briefly described in the literature. One mother with severe type A foodborne botulism breastfed her infant aged 8 months while acutely ill with cranial nerve palsies, weakness, and shortness of breath that required intubation and respiratory support for 2 weeks (*117*,*118*). Neither *C. botulinum* nor botulinum toxin were identified in her breastmilk, which was obtained for testing on the third day of her illness, 4 hours after she received trivalent ABE botulinum antitoxin. The infant was reportedly breastfed throughout the mother’s illness, including before the mother received antitoxin, and did not develop any signs or symptoms of botulism. The infant did not receive antitoxin, *C. botulinum* was not identified in the infant’s stool, and botulinum toxin was not identified in the infant’s stool or serum collected on the third day of the mother’s illness. Another study involved an infant aged 2 months who breastfed while the mother was acutely ill with botulism (*119*). The mother was reported to have died from type A botulism; however, the signs and symptoms she experienced were not specified. The infant did not develop any signs or symptoms of botulism. The study did not specify whether the infant received testing. Another infant aged 2 months breastfed without becoming symptomatic while the mother was acutely ill with type B botulism (*120*). The mother had cranial nerve palsies and generalized weakness and required a tracheostomy. The infant received antitoxin (timing of antitoxin not specified) and remained asymptomatic. The study did not specify whether the infant received testing.

The BAT package insert states that no data are available to assess the presence or absence of BAT in human milk, the effects on breastfed children, or the effects on milk production or excretion (*84*). BAT’s molecular weight (150,000 daltons) might prevent BAT from entering breast milk (*84*). Few infants have received BAT. One study found that an infant aged 10 days was treated with BAT without any known adverse events, and another study found that a neonate developed a low-grade fever within 1 hour of receiving BAT that continued intermittently for 72 hours (*37*,*121*).

### Recommendations

- Treat breastfeeding women in accordance with recommendations in the treatment section of these guidelines (see Treatment Considerations).
- If the mother continues to breastfeed, monitor the infant closely for signs and symptoms of botulism and for adverse events from BAT.
- Although the risk for acquiring botulism from the breast milk of mothers who have botulism and do not receive antitoxin treatment is unknown, clinicians and family members should be aware that botulism is a life-threatening illness and that the delay between a request for antitoxin to administration of the antitoxin is typically 1–2 days. Interruption of breastfeeding for this period would have minor to no consequences for the child but could affect maternal milk supply and lead to a serious breast infection. If the decision is made to temporarily stop breastfeeding, the mother should express her milk and throw it away until administration of the antitoxin. This should be done with support from a lactation specialist.

## Critical Care: Considerations During Shortages

With adequate critical care, especially intubation and mechanical ventilation when needed, almost all patients with botulism survive and eventually fully recover, even without receiving antitoxin. Clinicians should proactively manage and prioritize critical care to avoid shortages. Thorough planning can be used to anticipate several measures that, when implemented promptly during an outbreak, can help prevent or reduce critical care shortages. When a botulism outbreak is recognized, the extent of the outbreak should guide the mobilization of local, regional, and federal assets as quickly as possible on an appropriate scale. This might include early transfer of supplies and equipment (e.g., intubation supplies, bag-mask combinations, ventilators, sedatives, anesthesia machines, and transport ventilators) from regional or federal sources to the treating medical facilities. Because botulism is not contagious and patients are usually hemodynamically stable, moving patients to facilities with adequate resources might be critical during a large outbreak; proactive mobilization of transportation resources in such circumstances is important.

### Supportive Care

Because generalized paralysis can require prolonged intubation and mechanical ventilation, many patients are hospitalized for weeks to months and might be at risk for adverse events that complicate their care. Various recommendations and guidelines are available to help prevent serious complications such as catheter-associated urinary tract infections, pressure ulcers, DVT, and ventilator-associated pneumonias (*122*–*124*). Because of the similarities between Guillain-Barré syndrome and botulism, experience with caring for patients who have this more common disease might be helpful. Several reports highlight major issues that can complicate Guillain-Barré syndrome and multidisciplinary approaches to prevent them (*77*).

Because the complications of prolonged paralysis are well known to clinicians and general guidelines are available to help prevent complications, these guidelines focus on issues that might be unique to patients with botulism or that require additional attention. Because botulinum toxin is unlikely to cross the blood-brain barrier in humans, the toxin does not exert any direct effect on the central nervous system (*116*). Patients with botulism are typically alert and have no cognitive deficits unless they are hypoxic, are intoxicated from alcohol or illicit drugs (e.g., black tar heroin), are receiving sedatives, or have a secondary process resulting in decreased cognition. Facial paralysis, ophthalmoplegia, slurred speech, and inability to respond to requests because of muscle weakness might lead persons to think the patient has altered mental status or is comatose, although they actually are alert, aware, and listening and comprehending. The importance of understanding that patients with botulism are typically awake and alert despite their appearance is highlighted by one patient’s description of being unable to communicate with her health care providers initially and undergoing painful procedures (e.g., EMG and NCS) with no explanation from clinicians (*125*). One study systematically reviewed communication methods among conscious, critically ill patients who were receiving mechanical ventilation and provided an algorithm that might be used to optimize communication (*126*).

Patients with botulism and their families experience emotional distress because of the life-threatening nature of botulism, the unpredictable prognosis of the condition, the need for multiple medical procedures, and communication difficulties (*127*). Psychosocial support services have been associated with a reduction in feelings of helplessness and anxiety among patients and family members by the second week of hospitalization. Clinicians who have cared for patients with botulism have reported that music, massage therapy, and reading aloud were often beneficial to their patients (CDC, unpublished data, 2016). Patients with botulism often have autonomic dysfunction because of impairments in the parasympathetic nervous system, resulting in dry eyes, dry mouth, urinary retention, and ileus (*70*,*72*). However, anecdotal reports from a 2015 Ohio botulism outbreak linked to potato salad indicated that patients reported copious oral secretions, perhaps because of dysphagia and the decreased ability to swallow oral secretions (CDC, unpublished data, 2016).

### Recommendations

- Inform staff members that patients with botulism are typically cognitively intact.
- Establish a system to enable communication between the patient and health care providers. Explain procedures before performing them.
- Provide meticulous attention to bladder and bowel care and the prevention of complications, such as urinary tract infections, DVT, and pressure ulcers.
- Assess patients for anxiety and depression and provide psychological support as needed.
- Institute speech, physical, and occupational therapy as soon as possible.
- Consider music and massage therapy and asking family members or staff members to read aloud to the patient.
- Evaluate for and treat dry eyes and dry mouth; anticipate the possibility of copious oral secretions.
- Educate family members about botulism, and provide information about supportive care, treatment, and prognosis. Discuss psychosocial support resources that might be available to family members, and consider instituting support groups if multiple patients are hospitalized in the same facility or in nearby facilities.

## Conclusion

The systematic reviews and discussions during the forums and workshop identified evidence gaps in the diagnosis, monitoring, and treatment of patients with botulism. For clinical diagnosis, identification of clinical features that increase the sensitivity and specificity of clinical diagnosis could facilitate treatment decisions and clinical management. For laboratory diagnosis, the number of days that botulinum neurotoxin persists in the serum of the average patient with botulism is not well established. Better data on the persistence of toxin in serum would help ensure laboratory testing is conducted when warranted. Regarding monitoring neurologic and respiratory status, the optimal methods and frequency of assessment also are unclear. Identifying the optimal methods and frequency would ensure resources are used appropriately, which is especially important in contingency or crisis standards of care. From the treatment standpoint, the point in the course of illness at which botulinum antitoxin offers no further benefit has not yet been identified. Identifying this point might help determine a process for administering antitoxin during an antitoxin shortage. These evidence gaps remain because of the rarity of botulism and a lack of evidence that is more robust than case reports and case series. Prospective studies of patients with botulism might help address these gaps, providing clinicians and public health professionals with additional data on how to treat patients and prepare for and respond to botulism outbreaks.

## Footnotes

* The strength of botulinum antitoxin is quantified in terms of neutralization capacity, which reflects the amount of toxin that a dose of antitoxin can render inactive. The potency of botulinum toxin is quantified in units called the mouse intraperitoneal lethal dose_50_ (MIPLD_50_), the amount of toxin, specific for each serotype, required to kill one half of a group of mice injected intraperitoneally. The antitoxin’s neutralization capacity for each toxin type is the number of MIPLD_50_ that an amount of antitoxin (of that toxin type) can neutralize and is expressed in international units (IUs). One IU of antitoxin type A, B, C, D, or F neutralizes 10,000 MIPLD_50_ of the corresponding toxin; one IU of antitoxin type E neutralizes 1,000 MIPLD_50_ of toxin type E. The neutralizing IU for toxin type G has not been standardized.
